# Update on Potentially Zoonotic Viruses of European Bats

**DOI:** 10.3390/vaccines9070690

**Published:** 2021-06-23

**Authors:** Claudia Kohl, Andreas Nitsche, Andreas Kurth

**Affiliations:** Robert Koch Institute, Centre for Biological Threats and Special Pathogens, 13353 Berlin, Germany; NitscheA@rki.de (A.N.); kurtha@rki.de (A.K.)

**Keywords:** bats, virome, metagenomics, Issyk-Kul virus, SARS-like CoV, zoonoses, Zwiesel bat banyangvirus, Mammalian orthoreovirus, Lloviu virus

## Abstract

Bats have been increasingly gaining attention as potential reservoir hosts of some of the most virulent viruses known. Numerous review articles summarize bats as potential reservoir hosts of human-pathogenic zoonotic viruses. For European bats, just one review article is available that we published in 2014. The present review provides an update on the earlier article and summarizes the most important viruses found in European bats and their possible implications for Public Health. We identify the research gaps and recommend monitoring of these viruses.

## 1. European Bat Viruses 

Bat viruses have been gaining worldwide attention following the outbreaks of SARS-Coronavirus (CoV), SARS-CoV-2, Nipah virus, Hendra virus, and Ebola virus. Worldwide sequences of 12,476 bat-associated viruses are available at NCBI Genbank and DBatVir (accessed on 31 March 2021) [1,2]. The highest number of sequences is available from Asia (5225), followed by Africa (2728), North America (1889), Europe (1353), South America (1065), and Oceania (216). In comparison to Asia and Africa, the number of European bat viruses discovered seems low. As virus species richness is positively correlated with species richness and abundance, it is coherent that more viruses are discovered in the species-rich tropical regions [3,4]. Additionally, the prominent examples of zoonotic bat viruses have been emerging in Asia and Africa; this is consequential since the highest number of bat viruses was detected on these continents. European bat species are covered by species protection through the European Commission (http://ec.europa.eu/environment/nature/legislation/habitatsdirective, accessed on 22 June 2021) and through the Agreement on the Conservation of Populations of European Bats (www.eurobats.org, accessed on 22 June 2021); therefore investigative research requires special permission by local government bodies. This might contribute to the lower number of viruses detected in Europe and North America. Nevertheless, the viral richness discovered in European bats is high. 

The current SARS-CoV-2 pandemic is once more underlining the importance of viral discovery in bats. If we can come back to databases containing the sequences of the viral diversity in the respective hosts, it becomes more feasible to determine which measures need to be taken. This review aims to provide an overview on viruses discovered in European bats. In addition, we identify the research gaps, as data on critical factors necessary for an assessment of the zoonotic risk are rarely reported. For most of the viruses, data is unavailable on viral shedding of infectious virus, prevalence of the virus in the host population, abundance of hosts and habitat overlap with humans, identification of potential transmission routes, and data on shedding seasonality. We discuss the potential anthropozoonotic and zoonotic transmission between bats and humans and propose to further investigate certain bat viruses.

### 1.1. Bat Virus Discovery in Europe

**Figure 1:**
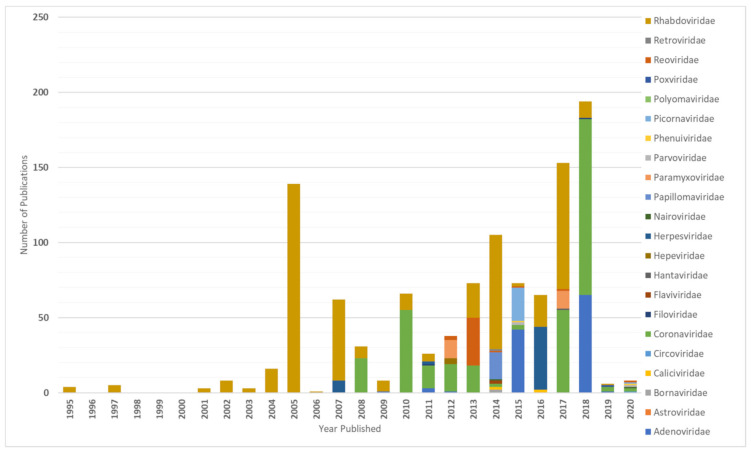
Number of published virus sequences over time, related to the respective viral family and order (DBatVir [1]).

**Figure 2:**
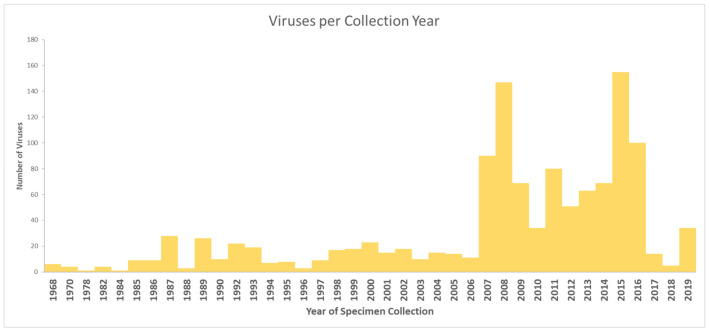
Number of published virus sequences by year of specimen collection (DBatVir [1]).

**Figure 3:**
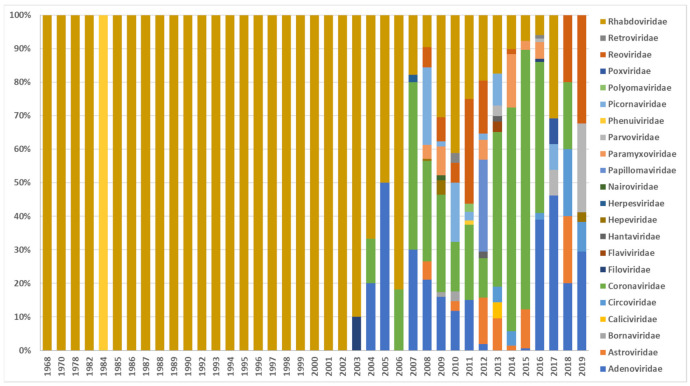
Published virus sequences by year of specimen collection, related to the respective viral family and order (DBatVir [1]).

**Figure 4:**
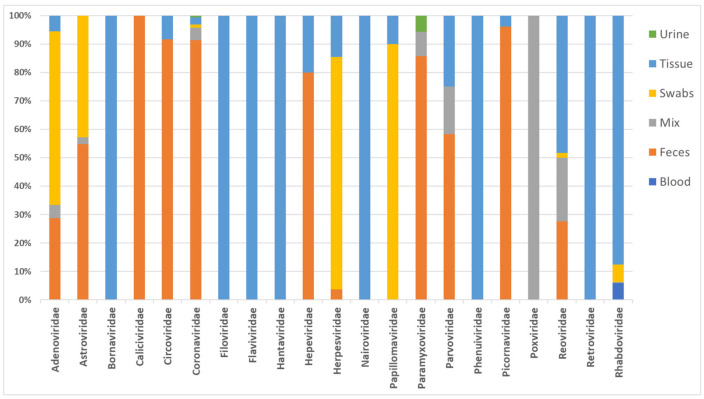
Specimen type used for virus detection related to the respective viral family and order (DBatVir [1]).

The first sequence of a European bat virus in the database was reported in 1995, and the oldest collected European bat specimens were from 1968 [1,5]. The greatest attention was paid to Rhabdoviruses before the virus discovery studies have been diversifying from 2007 on. Figure 1 shows the number of published virus sequences over time, related to the respective viral family and order. However, another criterion to determine virus discovery in Europe is the number of published viruses by year of specimen collection, as shown in Figure 2 that summarizes the number of published viruses by year of specimen collection. Figure 2 displays that the number of discovered viruses and, we assume, likewise the efforts in specimen collection had grown ten-fold in 2007. This increase in sample collection and virus discovery studies may be the result of the increasing recognition of bats as potential reservoir host of emerging viruses. Bats were confirmed as reservoir host of Hendra virus in 2000 [6], Nipah virus in 2001 [7], SARS-like CoV in 2005 [8], and Marburg virus in 2009 [9]. In addition, they were postulated as potential host of Ebola virus in 2005, MERS-CoV in 2012, and SARS-CoV-2 in 2020 which still has to be confirmed [10,11,12]. Figure 3 illustrates how the discovery of bat viruses has been diversifying from 2003 on, while virus discovery focused on Rhabdoviruses in bats until 2002. From 2003 on we see an increased discovery of CoV in European bats. On the one hand, this might be due to the fact that CoV (among other viruses) can be detected in feces samples and are therefore more accessible to research than other specimens (compare Figure 4). On the other hand, CoV are very abundant in bats and have a high tenacity, making them more likely to be detected compared to e.g., Paramyxoviruses. Moreover, the lack of data on negative tested bats raises difficulties to draw conclusions [13,14,15,16,17]. Another factor is the availability of bat species for examination. Bat species are very divergent in their roosting and migration behavior, making it difficult to collect specimens from some species and easy from others. In contrast to studies in other areas of the world, the European bats are strictly protected; thus bat sampling is more complicated and results in a potential underrepresentation of the number of bat viruses reported. An overview on viruses discovered by bat species in Europe is given in Table 1. It seems that most viruses were found in *Myotis* spp., *Pipistrellus* spp., and *Eptesicus* spp. Here also, without data on bats that were sampled but tested negative, it is hard to draw conclusions. 

Another reason for the generally increased detection of viruses could be the great progress in virus discovery methods during the same time-frame. While in the early years of virus discovery researchers had to rely on time-consuming cell-culture methods for virus detection, the “molecular evolution” was a game changer. Not only PCR, primer design, and capillary sequencing were becoming cheaper and thus widely available, also massive parallel sequencing methods were gaining attention. It was in 1994 when Canard and Safarti first published the baseline for Illumina sequencing technology [18]. In 2005 Margulies et al. published the massive parallel sequencing method of 454 sequencing [19]. In 2013 already, Roche shut down the 454 sequencing branch, as Illumina became market leader. Since 2014 portable sequencing via Oxford nanopore is on the rise [20,21]. Metagenomics and viromics have become standard applications in the virological research communities, leading to increased virus discovery results in European bats [15,22,23,24,25,26,27,28,29,30,31,32,33]. However, isolation of viruses is still the gold standard in virology for subsequent functional characterization and it will be very hard to replace this method. 

### 1.2. Viruses Detected in European Bats

**Figure 5:**
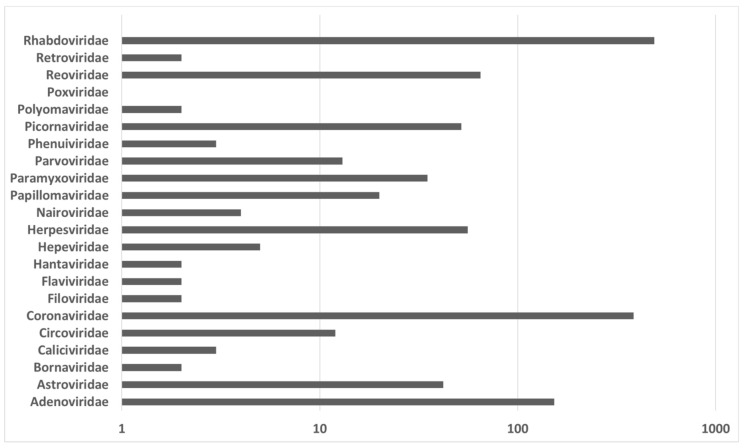
Number of viruses by family recorded for European bats in log scale (DBatVir [1]).

Until now, the database of bat viruses comprises 1353 entries for Europe (accessed on 31 March 2021) [1]. A summary of all entries (viruses vs. bat species) can be found in Table 1. Table 2 provides references, host bat species, and detection methods for all viruses found in European bats. Figure 5 displays the number of viruses by family recorded for European bats. The majority of viruses recorded in the database belongs to the families *Rhabdoviridae* and *Coronaviridae*. Since the first review on zoonotic viruses of European bats in 2014 [2] various novel viruses have been discovered. In the following section we focus on these viruses that in our opinion could possibly pose a zoonotic threat to humans. The full and up-to-date list of European bats can be accessed online at the Database of Bat-associated Viruses (DBatVir) [1].

#### 1.2.1. Coronaviruses

Numerous CoV have been detected in bats, most of which belong to the genus Alpha- and Betacoronaviruses [1,112]. The genus Alphacoronavirus hosts human-pathogenic strains (i.e., Human CoV 229E and NL63); however, in this review we focus on selected highly human-pathogenic Betacoronaviruses and their European bat virus relatives [112]. Several more comprehensive reviews on bats and CoV are available [113,114,115]. 

##### SARS-CoV

The first pandemic of the new millennium confronted the world from November 2002 until July 2003 with the severe acute respiratory syndrome in humans caused by a novel CoV (SARS-CoV, subgenus Sarbecovirus) [116,117]. The SARS-CoV pandemic spread from its origin, a wet-market in the Guangdong province in China, through 33 countries on five continents and resulted in more than 8000 infected humans of whom more than 700 eventually died [113,118]. Masked palm civets and bats were suspected as possible sources and reservoir species. Subsequently, numerous SARS-CoV-like viruses were detected in bats, some of which were able to use the ACE2 receptor crucial for human infection, without further modification [119,120]. A SARS-related bat CoV (HKU3) was isolated from Chinese horseshoe bats (*Rhinolophus sinicus*) [121]. Furthermore, Hu et al. identified several SARS-CoV-like viruses in 2017 in a colony of horseshoe bats in Yunnan province, China [122]. Three of these viruses display similar surface glycoprotein domains and are thus capable of using ACE2 as the receptor, and the authors assume that SARS-CoV originated from these viruses by recombination events and spillover [122,123]. Subsequently, a plethora of diverse CoV of distinct groups have been detected in various bat species around the world via molecular-biological techniques and virus isolation [114]. 

Numerous studies of European bats report the presence of Betacoronaviruses and several report SARS-like CoVs [27,42,50,56,57,60,61,64,65,66,67]. Remarkably, all SARS-like CoV were identified in bats of the family *Rhinolophidae*. In the UK, Slovenia, and Italy *Rhinolophus hipposideros* was the reported host of SARS-like CoVs with identities of >80% with SARS-CoV [27,57,64]. In Luxembourg, Italy, France, and Spain *Rhinolophus ferrumequium* was tested positive for SARS-like CoVs [51,54,60,67,68]. *Rhinolophus blasii* from Bulgaria was also found positive for SARS-like CoVs [61]. 

##### MERS-CoV

With the emergence of Middle East respiratory syndrome CoV (MERS-CoV, subgenus Merbecovirus) in 2012, another human-pathogenic CoV began spreading from the Arabian Peninsula [124], so far resulting in globally 2566 laboratory-confirmed cases of infection with MERS-CoV, including at least 882 deaths (WHO. Available online: https://www.emro.who.int/health-topics/mers-cov/mers-outbreaks.html, accessed on 9 April 2021). Dromedary camels were confirmed as reservoir host of MERS-CoV and a continuing source of transmission to humans [125]. However, it is widely assumed that MERS-CoV has initially originated from bats and was transmitted to dromedary camels >30 years ago [126]. This is further supported by the detection of MERS-CoV-related viruses, which share receptor usage for cell entry with MERS-CoV, in bats [127]. MERS-like CoV were detected in *Hypsugo savii* in Italy and in *Pipistrellus* spp. in Italy, the Netherlands, Germany, Ukraine, and Romania [57,63].

##### SARS-CoV-2

**Figure 6:**
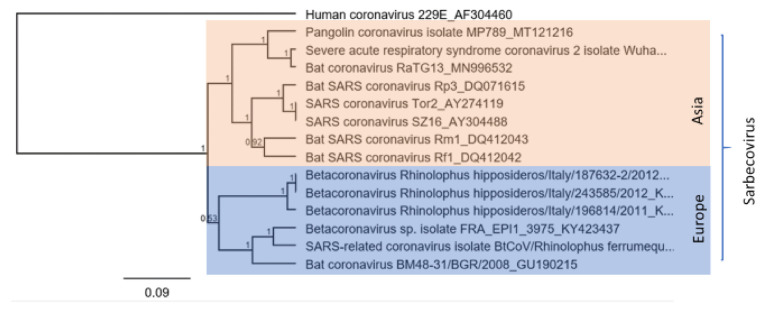
Phylogenetic reconstruction of European SARS-like Betacoronaviruses with SARS-like viruses and SARS-CoV viruses from Asia. Phylogenetic reconstruction was calculated based on a 392 nt long fragment of CoV available under the accession numbers mentioned in the tree. Calculations were performed using Clustal, MrBayes (GTR, 10 Mio, 10 percent Burn-in), visualization Geneious prime.

Since December 2019 another pandemic CoV, SARS-CoV-2 (subgenus Sarbecovirus), has been confronting the world [12]. SARS-CoV-2 became the seventh CoV known to be capable of infecting humans, so far resulting in globally 178,503,429 laboratory-confirmed cases of infection with SARS-CoV-2, including at least 3,872,457 deaths (WHO, 22 June 2021; https://www.who.int/emergencies/diseases/novel-coronavirus-2019, accessed on 22 June 2021). SARS-CoV, MERS-CoV, and SARS-CoV-2 are associated with severe diseases, while HKU1, NL63, OC43, and 229E cause rather mild diseases [128,129]. Several of the early cases of SARS-CoV-2 have been linked to the Huanan market in Wuhan, China [12,130]. Given the SARS-CoV pandemic and the resulting increased interest in bat CoV, a bat CoV (RaTG13, 96.2% id) detected in *Rhinolophus* *affinis* in the Yunnan province was quickly identified as the closest relative [12,122,131]. SARS-CoV and SARS-CoV-2 share 79.6% sequence identity only, although both viruses are using the ACE2 receptor for cell entry [12]. We have calculated a phylogenetic reconstruction for Asian and European SARS-like bat viruses in comparison to SARS-CoV, SARS-CoV-2, SARS-CoV from zibet and SARS-CoV-2 from pangolin (Figure 6). The European SARS-like viruses are clustering as a distinct sister clade to the Asian SARS-like bat viruses and SARS-CoV and SARS-CoV-2.

A related virus detected in bats cannot necessarily be considered as zoonotic. Few alterations in the SARS-CoV spike protein enabled binding to its host receptor ACE2; thus SARS-CoV became capable of infecting humans [132]. So far, the SARS-like CoV detected in European bats lack these alterations and are therefore not predicted to be capable of infecting humans [129]. However, at least two theories are being discussed about the proximal origin of SARS-CoV-2 and the way that SARS-like CoVs of the Yunnan province may have acquired ACE2 receptor usage: 1. Natural selection in an animal host by zoonotic transfer, in contrast to RaTG13 bat virus (the closest relative of SARS-CoV-2). Some pangolin CoV show a great similarity in the receptor-binding domain, although neither a bat nor a pangolin virus has been detected so far that would be sufficiently similar to SARS-CoV-2 to serve as a progenitor virus [129,133,134]. 2. Natural selection in humans following zoonotic transfer: a progenitor virus would have jumped into the human host, adapted, and acquired the necessary genomic features during human-to-human transmission [129]. Taking these theories into account and given the present worldwide pandemic, it becomes reasonable to monitor viruses of concern throughout European bat populations. The diversity of CoV in bats seems to be immensely high. Although numerous CoV have already been identified, the real diversity (also of possible progenitor viruses) and the potential risks remain unclear. 

#### 1.2.2. Bat Filovirus

The family *Filoviridae* comprises six genera, four of which (*Marburgvirus*, *Ebolavirus, Dianlovirus* and *Cuevavirus*) are associated with bats as either confirmed or suspected reservoir host species [112]. Marburg virus (MARV) was isolated in 1967 in Marburg, Germany. It became apparent that the 32 persons who contracted MARV (of which seven died) handled specimens from vervet monkeys (*Cercopithecus aethiops*) imported from Lake Victoria, Uganda [135,136]. The patients revealed flu-like and gastrointestinal symptoms. Later on, 25 percent of them developed signs of hemorrhagic diathesis and bled from all body orifices and needle punctures [136]. In consecutive experimental infections with MARV, the vervet monkeys showed clinical symptoms and died, leading to the assumption that they were not the natural MARV reservoir hosts [137]. Subsequent studies investigated different animals as potential reservoir hosts before MARV was successfully isolated from *Rousettus aegyptiacus* and the bat reservoir hypothesis was proved correct [9,138]. Consecutive cases of MARV infections in humans were sporadically connected to mineworking or tourist visits to mines inhabited by bats [139,140,141,142].

The genus Ebolavirus comprises six distinct species four of which cause severe hemorrhagic fever similar to MARV in humans and primates (*Bombali ebolavirus*, *Bundibugyo ebolavirus*, *Reston ebolavirus*, *Sudan ebolavirus*, *Taï Forest ebolavirus,* and *Zaïre ebolavirus*) [112,143,144]. With the exception of Reston ebolavirus, all ebolaviruses were detected in Africa. Ebolavirus was named after the Congolese Ebola river and first emerged in Zaïre (nowadays Democratic Republic of the Congo; DRC) in 1976 and simultaneously in the Sudan [145]. During the search for the reservoir host, bats were increasingly suspected and examined [137]. In 2014, Zaïre ebolavirus strain Mayinga (ZEBOV-May) emerged in Guéckédou within the prefecture of Nzérékoré, Guinea [146]. Later on ZEBOV-May spread to Liberia, Sierra Leone, Nigeria, and Mali, resulting in the largest outbreak of ebolavirus reported so far, with 28,616 laboratory-confirmed cases and 11,310 deaths (https://www.cdc.gov/vhf/ebola/history/2014-2016-outbreak/index.html, accessed on 23 April 2021). It is assumed that the whole epidemic started with a single zoonotic transmission event to a 2-year-old boy playing in a hollow tree housing a colony of insectivorous free-tailed bats (*Mops condylurus*) [147]. RNA of a recently discovered ebolavirus, Bombali ebolavirus, was first detected in *Mops condylurus* and *Chaerephon pumilus* in Sierra Leone, the prefecture of Nzérékoré, Guinea, and Kenia [148,149,150]. The potential of Bombali ebolavirus to cause diseases in humans remains unknown. In 2015, Reston ebolavirus was detected in a bat (*Miniopterous schreibersii*) in the Philippines [151]. 

The genus Dianlovirus comprises a single species, Měnglà virus (MLAV), identified in lung tissues of *Rousettus* spp. and *Eonycteris spelaea* in Yunnan province, China [152].

The genus Cuevavirus also comprises a single species, Lloviu virus (LLOV). LLOV was detected in suddenly declining colonies of Schreiber’s bats (*Miniopterus schreibersii*) in France, Spain, and Portugal in 2002 [69]. LLOV detection was limited to animals that showed signs of viral infection. Healthy co-roosting bats (*Myotis myotis*) were investigated but LLOV was not detected. LLOV is distinctly related to Filoviruses found in African bats (EBOV) and was classified in 2013 as type species of the novel genus Cuevavirus [112]. In 2015, a study by seroprevalence demonstrated wide circulation of LLOV antibodies in Schreiber’s bats in Spain [153]. After mass die-offs of Schreiber’s bats in Hungary (2013, 2016, and 2017) LLOV was confirmed in Schreiber’s bat carcasses presenting with hemorrhagic symptoms [28]. Schreiber’s bats are reported by banding data as a seasonally migrating species with flight distances ranging from a few hundred to 800 km (section migration). Schreiber’s bats are distributed in distinct lineages throughout Oceania, Africa, southern Europe, and South-East Asia [154]. Given that LLOV was found in Spanish and Hungarian Schreiber’s bats, there may also be some gradual circulation between colonies of Schreiber’s bats in between Spain and Hungary. As most Filoviruses are described as highly pathogenic for humans, the occurrence of LLOV should be carefully monitored by banding studies and surveys on viruses of Schreiber’s bats to assess these findings. 

#### 1.2.3. Bat Flaviviruses

The genus Flavivirus comprises a variety of arthropod-borne human-pathogenic viruses (Arboviruses) with a high impact on global health (i.e., Dengue virus, Zika virus, Yellow fever virus, Tick-borne encephalitis virus, West Nile virus). In 1970, West Nile virus (WNV) was detected and isolated from bats (*Rousettus leschenaultii*, Lesser Short-nosed Fruit Bats, Lesser Sheath-tailed Bats, and Thai Horseshoe Bats) in India, Malaysia, and Mexico [155,156,157]. Subsequent to the epizootic emergence of WNV in the USA, Mexico, and Canada, studies on amplification hosts (other than birds) were performed. Although low levels of antibodies to WNV were detected in *Eptesicus fuscus* and *Myotis septentrionalis* from Illinois, New Jersey, and New York, USA, an experimental infection of North American *Eptesicus fuscus* and Mexican *Tadarida brasiliensis* bats resulted in the conclusion that bats were unlikely to serve as amplification hosts of WNV [158,159,160]. Recently, Zika virus was detected in *Artibeus jamaicensis* in Mexico [157]. In addition to these cases, a variety of Flaviviruses was isolated from or detected in bats in Asia, the Americas, and Africa; overall seroprevalence studies indicated a low prevalence of Flaviviruses in the bats’ sera and experimental infection showed signs of poor replication [161,162,163,164,165,166]. The poor replication in the host bats’ tissues upon experimental infection conflicts with the theory that bats are involved in the sylvatic cycle of arboviral Flavivirus transmission [167]. 

Usutu virus (USUV) belongs to the Japanese encephalitis serocomplex of Flaviviruses [70]. Migratory birds and mosquito vectors (mainly *Culex* spp.) are assumed to play an important role as amplification hosts and in introducing USUV into new areas, as recently shown for Europe where USUV has been causing epizootics among wild birds and Usutu fever in humans [168]. In 2013, two dead-found bats (*Pipistrellus pipistrellus*) were investigated in the south-west of Germany and USUV was detected in the brain of both individuals [70]. Full genomes were sequenced and showed 99.3 percent identity (nt) to a bird-derived strain BH65/11–02–03 from Germany [70]. The authors assume that the bats may act rather as coincidental hosts than as reservoirs of USUV. 

#### 1.2.4. Bat Bunyaviruses

The order *Bunyavirales* comprises twelve families of whom five are associated with severe diseases in humans (*Arenaviridae*, *Hantaviridae*, *Nairoviridae*, *Peribunyaviridae,* and *Phenuiviridae*) [112]. 

##### Hantavirus

In humans Hantaviruses cause hemorrhagic fever with renal syndrome (HFRS) in Asia and Europe and Hantavirus cardiopulmonary syndrome (HCPS) in the Americas [169]. Hantavirus sequences have been detected in several bat species of Sierra Leone, Vietnam, Brazil, Côte d’Ivoire, China, Myanmar, Gabon, and Ethiopia [1,170,171,172,173,174,175]. In Europe a novel Hantavirus (Brno virus) was detected in common noctule bats (*Nyctalus noctula*) in the Czech Republic [71]. This virus is related to Longquan virus (LQUV) detected in *Rhinolophus* spp. in China [172]. These viruses are only distantly related to other Hantaviruses described so far. 

##### Phenuivirus

Within the family *Phenuiviridae* there are 19 genera. Viruses of the genus Phlebovirus are transmitted by sandflies and mosquitoes (Phlebotomus group) or ticks (Uukuniemi group) and several were linked to human diseases [112]. Toscana virus (TOSV) and Rift Valley fever virus (RVFV) are the most prominent examples. Toscana virus is transmitted by sandflies and ranges among the three most prevalent viruses causing meningitis in the Mediterranean (in particular Italy) during the warm season [176]. RVFV is transmitted to humans either vectorially through mosquito bites or by direct contact to infected tissue [177]. The disease phenotype of RVFV in humans ranges from unapparent to severe courses of hemorrhagic fever and meningoencephalitis [178]. RVFV has been isolated from bats of the species *Micropteropus pusillus* and *Hipposideros abae* in the Republic of Guinea [178]. The only reported Phenuivirus in Europe associated with bats was Toscana virus from the brain of one *Pipistrellus kuhlii* bat in Italy, although doubts have arisen in this early finding which might be due to possible cross-contamination [1]. Two novel Phenuiviruses were recently identified in German bats by metagenomics from *Eptesicus nilssonii* tissue: Bavarian bat lalavirus (BblV, *Pipistrellus nathusii*) and Munich bat lalavirus (MblV, *Pipistrellus nathusii*). BblV and MblV are distantly related to other members of the Uukuniemi group [15].

Within the family *Phenuiviridae*, viruses of the genus Bandavirus have caused febrile infections, encephalitis, and severe fevers with fatal outcome in humans. Recently, two novel tick-borne Phenuiviruses (Severe Fever with thrombocytopenia virus (SFTS), recently renamed Huaiyangshan banyangvirus and more recently renamed Dabie bandavirus, and Heartland virus (HRTV)) were detected and characterized. SFTS was initially reported in 2011 in the Henan and Hubei provinces, China. Patients developed hemorrhagic fever, thrombocytopenia, leukocytopenia, and multi-organ dysfunction with an initial case fatality rate of 30 percent [179,180]. By then, the etiological virus was isolated from patients’ blood and *Haemaphysalis longicornis* and *Rhipicephalus microplus* ticks throughout China, South Korea, and Japan [181,182]. Similar symptoms were recognized in two men from Missouri, USA. The respective virus, named Heartland virus (HRTV), was isolated in 2012 from patients’ blood and *Amblyomma americanum* ticks collected in the field [183,184]. Despite the identification of ticks as vectors for SFTS and HRTV, the reservoir hosts of the viral pathogens remain unknown. In 2014, Malsoor virus, a related Bandavirus, was isolated from *Rousettus leschenaultii* in India [26,185].

A novel Bandavirus strain was recently identified in German bats by metagenomics from bat tissue: Zwiesel bat banyangvirus (ZbbV, *Eptesicus nilssonii*) [15]. The German ZbbV is closely related to Malsoor virus. Both viruses cluster monophyletically with the genus Bandavirus which comprises SFTS and HRTV capable of causing severe diseases in humans [26,185]. 

##### Nairovirus

The family *Nairoviridae* contains the genus Orthonairovirus, named after the Nairobi sheep disease orthonairovirus (NSDV) species [186]. NSDV and other members of the genus, like Crimean Congo Hemorrhagic Fever virus (CCHFV), Dugbe virus, and Ganjam virus, are highly pathogenic to animals and humans [187]. Orthonairoviruses are often transmitted by ticks. As the viruses were not detected in wild ruminants or other animals in enzootic areas, the vertebrate reservoir host of these viruses remains unknown. Several Nairoviruses with unknown zoonotic potential have been detected in bats from Senegal, Uganda, Zambia, and French Guiana. A seroprevalence study conducted on African bats (*Rousettus aegyptiacus*, *Coleura afra*, *Hipposideros caffer*, *Miniopterus inflatus*, and *Hipposideros gigas*) found first evidence of a widespread prevalence of CCHF-like viruses within these species [188].

In Europe, a bat Nairovirus, Ahun Nairovirus, has been detected in lung tissues of one *Pipistrellus pipistrellus* and one *Myotis mystacinus* in France [22]. Phylogenetically, Ahun Nairovirus appears as a new clade distinct from other Orthonairoviruses [22]. Further three Nairoviruses have been detected in German bats by metagenomic sequencing: Berlin bat Nairovirus (BbnV, *Pipistrellus pipistrellus*), Wittenau bat Nairovirus (WbnV, *Pipistrellus pipistrellus*), and Issyk-Kul virus strain PbGER (*Eptesicus nilssonii*) [15,25]. BbnV is related to Sapphire II virus (Id 85% nt) and clusters with the Dera Ghazi Khan genogroup usually associated with birds and not described as human pathogenic [189]. WbnV is phylogenetically distantly related to Avalon virus (Id 71% nt) which was initially isolated from ticks in France [190,191]. Both cluster monophyletically with the Sakhalin genogroup; viruses of these genogroups have not been described before to be associated with bats [190,191]. Issyk-Kul virus strain PbGER is very closely related to Issyk-Kul virus LEIV315K (Id 95% nt), both clearly allocated within the Keterah genogroup [25]. Issyk-Kul virus was first isolated in 1970 from *Nyctalus noctula* bats in Kyrgyzstan, Tajikistan, and Kazakhstan [192,193]. *Eptesicus nilssonii* is a common bat distributed throughout Asia and Europe (including the polar regions). In Scandinavia they are even the most frequent bat species. They are dependent on humid habitats in close proximity to fresh water. In winter, they hibernate on heated attics and in wall claddings of human dwellings. For Issyk-Kul virus sporadic febrile outbreaks in humans are described with headache, myalgia, and nausea. It is assumed that Issyk-Kul virus is transmitted by tick bites and exposure to bat feces and urine [192,193]. These findings show for the first time the abundance of Nairoviruses in Europe and within this species. 

#### 1.2.5. Bat Reoviruses

The family *Reoviridae* is divided into the subfamilies *Sedoreovirinae* and *Spinareovirinae*. 

Within the *Sedovirinae* the genera Orbivirus and Rotavirus are of public health importance, as they comprise bluetongue virus and rotavirus types A, B, and C. Bat Orbiviruses were detected in China, Uganda, Guinea, Nigeria, Bangladesh, and Germany. In Germany, the Orbivirus was detected in a common noctule bat (*Nyctalus noctula*) [15]. This strain shares similarity with the yet unpublished Bat Orbivirus from China (AccNo. MH144554.1) (Id 81% aa) and Sathuvachari virus first isolated in India in 1963 [194]. Bat Rotaviruses are described in bats from China, Kenya, Gabon, Korea, and Cameroon. In Europe, numerous bat Rotaviruses were also discovered in bats from France (*Myotis myotis*), Germany (*Pipistrellus pipistrellus*), Bulgaria (*Rhinolophus blasii*, *R. euryale*), and Serbia (*Miniopterus schreibersii*) [15,22,32,85]. All strains, excluding the strain from Serbia, were allocated to Rotavirus species Rotavirus type A. The zoonotic potential of these bat Rotaviruses related to group A has yet to be determined.

The subfamily *Spinareovirinae* comprises among others the genera Coltivirus and Orthoreovirus, both associated with diseases in humans. A Coltivirus was isolated from *Chaereophon aloysiisabaudiae* in Côte d’Ivoire [195]. Orthoreoviruses were isolated from fruit bats in Australia (Nelson Bay virus) and Malaysia (Pulau virus) [196,197]. In 2007, Melaka virus (closely related to Pulau virus) was isolated from human patients in Malaysia and a zoonotic bat-borne transmission was assumed [198]. Since then five additional Orthoreoviruses (Xi-River, Kampar, Sikamat, HK23629/07, and Broome virus) have been isolated from fruit bats [199,200] or from humans with assumed contact to bats [201,202,203]. Three Orthoreoviruses were detected and several ones isolated from German bats (*Plecotus auritus*, *Myotis mystacinus*, *Pipistrellus pipistrellus*, *Pipistrellus nathusii*, *Pipistrellus kuhlii,* and *Nyctalus noctula*) [82]. Further 19 Orthoreoviruses in *Myotis kuhlii*, *Rhinolophus hipposideros*, *Tadarida teniotis,* and *Vespertilio murinus* were detected in Italy [83]. A close relationship of the strains from Germany and Italy was revealed to the genus Mammalian Orthoreovirus (MRV). In particular, they showed a high identity to an Orthoreovirus obtained from a dog (strain T3/D04) with hemorrhagic enteritis in Italy and an MRV isolated from a hospitalized child with acute gastroenteritis (strain SI-MRV0) in Slovenia [2,82,83,204,205]. The causative agent of the latter displayed high identity (ranging between 98.4% and 99.0% nt in the respective segments) to bat MRV (T3/Bat/Germany/342/08) isolated from *Plecotus auritus* in Germany [2,82,205]. These findings indicate a human-pathogenic potential for the MRV strains in European bats, and especially for strain T3/Bat/Germany/342/08. Interestingly, no contact was reported between the infected child and bats, but contact to a domestic dog was assumed [205]. In a second case a child with primary immunodeficiency was reported to be persistently infected with an MRV with very close relationship to the mentioned bat MRVs [206]. Further studies were conducted, elucidating the prevalence of potential zoonotic MRV strains in Slovenian and Italian bats [33,84,207]. The retrospective survey of Slovenian bat samples from 2008 to 2010 and in 2012 finally confirmed the occurrence of strain SI-MRV0 in the Slovenian bat populations and thus the zoonotic potential of bat-borne MRVs [84,205]. The isolated MRV could facilitate seroprevalence studies in humans which should be initiated to examine the prevalence of specific antibodies to bat MRVs in Slovenia, Italy, and Germany to further characterize their zoonotic potential. 

#### 1.2.6. Rhabdoviruses

Rhabdoviruses of the genus Lyssavirus are harmful and truly zoonotic agents, inevitably causing the death of unvaccinated humans if not treated in time before the onset of the rabies disease [208]. The genus Lyssavirus comprises 17 distinct species only two of which (Mokola virus and Ikoma Lyssavirus) most likely originated in bats [2,3]. The reported total number of human fatalities in Europe is low (*n* = 2–5 since 1963), even though bat-transmitted Lyssaviruses (by bat biting and scratching) have a case fatality rate of virtually 100 percent [208,209,210,211]. All so far described hosts of European bat Lyssaviruses (EBLV-1 and EBLV-2) are synanthropic, hence sharing their habitats with humans [210]. EBLV-1 was detected in *Eptesicus serotinus* and *E. isabellinus* in Europe, both living in buildings, roofs, and attics usually in the southern regions of Europe (*E. serotinus* until 55° N, *E. isabellinus* in southern Portugal), and male bats are reported to co-roost with multiple bat species [212]. EBLV-1 was also detected in *V. murinus, M. schreibersii*, *M. myotis*, *M. nattereri*, *R. ferrumequinum,* and *T. teniotis*. It has not yet been determined whether these bat species constitute accidental hosts infected by spillover from co-roosting *E. serotinus* species or whether they are additional reservoirs [92,93,101,108,213].

Two human cases described by Johnson et al. were confirmed to be infected with EBLV-2 which is prevalent in European *M. daubentonii* and *M. dasycneme* [101,208]. *M. daubentonii* is prevalent in north-eastern Europe and is frequently found co-roosting with *P. pipistrellus* and *M. nattereri*, whereas *M. dasycneme* is found throughout Europe and in the Mediterranean, co-roosting with *M. capaccinii*. So far, none of the co-roosting bats were reported to carry EBLV-2 [212]. However, spillover transmission to other animals (stone marten, sheep, and cat) was described for EBLV-1 [96,214,215].

The diversity of known European bat-associated Lyssaviruses has expanded. In 2003, West-Caucasian Bat Virus (WCBV) was isolated from *Miniopterus schreibersii* [107]. In 2011, Lleida Bat Lyssavirus (LLEBV) was detected also in *Miniopterus schreibersii* bats in Spain and later on in France [105,106]. Bokeloh bat Lyssavirus (BBLV) was identified in *Myotis nattereri* in Germany, France, and Poland [96,99,100]. Most recently, Kotalahti Bat Lyssavirus (KBLV) was detected in *Myotis brandtii* in Finland [86,104]. The rather novel BBLV and, tentatively, KBLV are (like EBLV-1 and EBLV-2) members of the phylogroup I Lyssaviruses. Several more comprehensive reviews on bats and bat Lyssaviruses are available [93,94,101,108,209].

#### 1.2.7. Other Novel European Bat Viruses

##### Caliciviruses

The first detection of Caliciviruses in European bats (*M. daubentonii*, *E. serotinus,* and *M. alcathoe*) was published in 2014 [45]. Fecal samples of Hungarian bats were screened by RT-PCR. While strain BtCalV/M63/HUN/2013 segregated with other viruses of the genus Sapovirus, the remaining two strains (BtCalV/BS58/HUN/2013 and BtCalV/EP38/HUN/2013) were unique and could not be classified to one of the already existing genera of Caliciviruses [42].

##### Parvoviruses

Metagenomic profiling of bats from Croatia, Germany, and Hungary resulted in the detection of several bat Parvoviruses [15,23,29]. In the Hungarian and German bats, sequences of bat Bufaviruses were identified [15,29]. The Hungarian Bufaviruses discovered in *M. schreibersii* were found to be phylogenetically related to the recently described human-pathogenic Bufaviruses, causing acute and severe diarrhea in children in Burkina Faso and Bhutan [29,216,217].

##### Picornaviruses

Bat Picornaviruses were identified in several bat species in Luxembourg, Germany, Spain, Romania, Hungary, and Italy [15,30,31,79]. Drexler et al. showed that bats harbored evolutionarily ancestral strains of Hepatoviruses [79]. Picornaviruses detected by metagenomics in German bats were related to King virus, Tetnovirus, and Hubei Picornavirus of invertebrates (id 66.0–99.0 percent nt) [15]. The Hungarian strain is highly divergent from other bat-derived Picornaviruses of the Sapelovirus genus [31]. The strain from Italy is distantly related to a bat Aichivirus [30]. All these findings support the idea of a possible ancestral origin of Picornaviruses in bats.

##### Polyomaviruses

Recently, bat Polyomaviruses were detected in Hungarian *Rhinolophus* bats [91]. These viruses were closely related to Polyomaviruses of Chinese and African horseshoe bats, suggesting a co-divergence of bat Polyomaviruses with their hosts during their evolutionary history [80]. 

##### Poxviruses

Hypsugopoxvirus (HYPV), a novel poxvirus, was isolated from *Hypsugo savii* in Italy [81]. HYPV is related to Eptesipoxvirus detected in *Eptesicus fuscus* in the USA [81], both viruses belonging to the *Chordopoxvirinae* subfamily genus Vespertilionpoxvirus.

## 2. Ecological Factors

Bats are the second largest order of mammals and compose about 20 percent of all extant mammals in the world [218]. They are the only mammals capable of active wing beat and flight, allowing them to migrate over vast distances. In summer, they can use torpor to reduce their body temperature in between ambient temperatures and the usual 37 °C, in winter they hibernate to save energy. It is important whether bats are long-distance migrants or sedentary species when investigating the respective colonies regarding zoonotic virus transmission. Furthermore, the possible effect of climate change on species richness and abundance of European bat species needs to be considered. This section will provide a short overview on the migration behavior and possible effects of climate change on European bat species.

### 2.1. Migration

The International Union for Conservation of Nature (IUCN) lists 53 bat species that inhabit the European continent, some of which are threatened with extinction on the population level and are hence protected under the IUCN Red List of Threatened Species and the Convention on the Conservation of Migratory Species of Wild Animals (CMS). All bats in Europe, also the fruit bat *Rousettus aegyptiacus* (inhabiting Cyprus), use echolocation to navigate. Numerous bat species migrate over vast distances while others are rather territorial. Hutterer and Ivanova summarized the available data on migration behavior of European bats based on 7366 migration routes recorded by banding [219]. 

They allocated the bats in three groups, sedentary species (up to 100 km of movement), seasonally migrating species (up to 800 km) and long-distance migrants (up to 4000 km) (Table 3) [219]. 

### 2.2. Climate Change

European bat species can be allocated to either one of three biogeographical groups, the Mediterranean, the Temperate, and the Boreal zone [220]. Current hotspots of European bat diversity are mainly located in the southern European peninsulas and in southern France [220]. Bat species hotspots of the Boreal group are located at the very northern end of Europe and these species are rarely found in southern Europe. Bat species of the Temperate group inhabit Central Europe and the United Kingdom. Even though the Temperate group is not the species-richest group, it is clearly the most widespread group in Europe [220]. Rebelo et al. modelled the effects of climate change on bat populations in the Boreal, Temperate, and Mediterranean zone [220]. They conclude that bats of the Boreal zone will face serious challenges to their survival by the end of the century. Depending on the model, the Temperate group will either increase species richness or face extinction in Central Europe. However, in every model used, the bats of the Temperate group will disappear from southern Europe. For the Mediterranean bats, the models predict that Central Europe will become highly suitable for the richness of Mediterranean bats in the future, while they will disappear from the Mediterranean zone. This theory is further supported by studies combining acoustic transect bat identification and modeling [221]. 

Another model by McCain found previously that the abundance of bats seems to be positively correlated with species richness; this suggests that bat species richness may also be related to productivity [222]. This means the more species are present in a selected region, the higher is the overall abundance of bats. All of the European bat species are protected by the Eurobats initiative as they are threatened by climate change, land-use changes, habitat loss, degradation, and wind turbines [62,223,224]. The latter might be connected to nocturnal insect migration and therefore also be affected by climate change [62].

Boyles et al. considered bats to be among the most economically important non-domesticated animal groups because of their important ecological roles as top predators and pollinators. Subsequently, in regions of bat diversity loss through climate change, the insect pest abundance would increase and pollination of food plants would be reduced [225]. 

## 3. Risk Factors

### 3.1. Zoonotic and Anthropozoonotic Transmission 

The assessment of the risk of zoonotic spillover of bat-borne viruses is of major importance for public health [226,227]. One important point is the aspect of climate change and how it affects the European bat populations. This is described in the “ecological factors” section. A study investigating the spatial hotspots of land-use changes in Europe from 1990 to 2006 found increased harvest on stable forest areas in central and northern Europe compared to the Mediterranean and western Europe [228]. Increased deforestation and urbanization within a host distribution has been shown to be positively correlated with the number of zoonotic viruses in a species [4,226]. By shifting bat populations northwards, the whole ecological system may be impacted and possible consequences in virus dynamics have to be monitored. Bat species predominantly abundant in southern Europe are suspected to be reservoirs of potentially zoonotic viruses (e.g., *Miniopterus schreibersii*, LLOV; *Rhinolophus ferrumequium*, SARS-like CoV) and would, according to climate models, thus be directly affected by climate change. 

#### 3.1.1. Could Spillover Be Facilitated by Bat Handling and Virus Research?

Bat research is not limited to virus discovery. Many disciplines study bats as one of the most special order of mammals. They are the subject of multifaceted studies investigating among others their bacteria, immunology, behavior, conservation, ecology, migration, echolocation, and evolution. They serve as model for e.g., the development of bionic aerodynamics and even mobility aid for the blind [229,230]. For all of these reasons and beyond, people have been handling bats for decades. Regarding risk assessment for bat viruses, we have to keep in mind how much (research) contact between humans and bats there is already and has not been reported so far to cause zoonotic spillover events. It is important to point out that zoonotic spillover is, to our knowledge, an extremely rare event that can usually only be evaluated retrospectively. However, generally the only people who could be exposed to a possible risk are those in direct contact with bats, their excretions, or their virus isolates (e.g., volunteers, bat workers, veterinarians, wildlife biologists, and also virologists). As we have no reports on any zoonotic virus transmission from bats to humans in Europe besides Lyssaviruses and Reoviruses, one could assume that these events would also be very rare in the future. 

In the context of the origins of the SARS-CoV-2 pandemic the question has arisen if the examination of bat hosts will facilitate virus emergence. Investigation, whether invasive or non-invasive, is stressful for the bats. A study investigating the stress-induced hypothermia (SIH) of silver-haired bats found that SIH is effected by capture and handling of the bats [231]. Following both the episodic shedding hypothesis and the transient epidemics hypothesis, it is assumed that for *Pteropid* bats stress can result in higher virus-shedding rates, as was already shown for Hendra virus and Nipah virus [232,233,234,235]. If this were applicable to European bat species, stress-triggered virus shedding would still not start immediately during bat handling but might be more important in the case of volunteers handling bats in nursery stations. However, it has yet to be determined to which degree insectivorous species are sensitive to stress in regard to episodic shedding. Even if bats wild-captured and released during investigations reacted later on with increased viral shedding rates, the risk of bat-to-human contact for the individual bat is negligible. 

Bringing samples to the lab and propagating bat virus creates possibilities of human–bat–virus interaction that would most likely not have occurred in nature. It is unlikely for laboratory workers to get infected by a virus in the laboratory, although lab accidents are reported. Following the SARS-CoV epidemic, three possible accidental laboratory-acquired infections were reported in Singapore, Taiwan, and China [236,237,238,239]. However, it is difficult to quantify lab-acquired infections because there is no systematic reporting system [240]. Wurtz et al. summarized the occurrence of laboratory-acquired infections around the world in BSL-3 and BSL-4 laboratories [240]. They identified human error to be the predominant cause of laboratory-acquired infections. In turn, this illustrates the effectiveness of the technical measures that are already in place. Human error in handling infectious specimens cannot be completely prevented, but the risk is minimized by conducting and observing biosafety training and creating an error management culture. To conclude, bat handling and bat virus research will most likely not lead to the introduction of viruses into the human population. Moreover, after individual laboratory infections there are no reports of any widespread laboratory-acquired infections. All reported infections were contained immediately. The WHO investigated the origins of the SARS-CoV-2 pandemic and concluded that it is extremely unlikely that a laboratory would have represented the origin of the pandemic [241]. They report that all three of the laboratories in Wuhan working with CoVs had high-quality biosafety level facilities that were well managed [241]. The benefit of researching bats and their pathogens by far exceeds, in our opinion, the risk of zoonotic spillover, as it entails the development of vaccines and therapeutics and allows for the thorough understanding of virus evolution and disease.

#### 3.1.2. Anthropozoonoses

Vice versa, especially during the current pandemic, we also have to discuss the possibility of anthropozoonoses. Human-to-animal transmissions of SARS-CoV-2 have already been described for minks, cats, and dogs [242,243,244]. In Denmark and the Netherlands, infected minks on mink farms developed respiratory disease with typical signs of viral pneumonia and were able to transmit the virus among each other and back to humans [242,245,246]. The source of infection pointed to humans as the initial source of infection based on genetic information and as no other connection was found between outbreaks on several farms [242,245,246]. It became apparent that mink farms can serve as reservoir of SARS CoV-2 and available SARS CoV-2 vaccines are less efficient in the mink-derived strain, thus resulting in the culling of 17 million minks in Denmark [247]. In addition, human-to-feline transmission of SARS CoV-2 was described for domestic cats as well as lions and tigers at the Bronx Zoo in New York, USA [243]. Occasional infections of dogs are also described [244,248]. Should an infected person come into contact with bats, for instance during field work in a bat cave, it cannot be ruled out that there is also a small potential for anthropozoonotic transmission. To elucidate whether bats are susceptible to a SARS-CoV-2 infection, experimental infection studies were conducted. A transmission study with SARS-CoV-2 in fruit bats (*Rousettus agyptiacus*) assumed transient infections after intra-nasal infection of nine bats with 10 × ^5^ TCID_50_ of SARS-CoV-2 [249]. Three native “contact bats” were added 24 h after infection, with one of three “contact bats” tested RNA positive for SARS-CoV-2, although no antigen or live virus was detected in any of the internal organs [249]. This is conclusive with an infection study in which *Rousettus aegyptiacus* bats were infected intranasally with a SARS-like CoV (WIV1-CoV), resulting in no signs of viral replication in the bats’ tissues [250]. Another study, experimentally challenging *Eptesicus fuscus* with SARS-CoV-2 in the US, did not find any evidence of successful viral replication in these bats [251]. As already described in the section “Viruses of European Bats,” SARS-CoV-like viruses were only detected in bats of the family *Rhinolophidae.* So far, the bat CoV closest related to SARS-CoV and SARS-CoV-2 were detected in *Rhinolophus sinicus* and *Rhinolophus affinis* in China, respectively [131]. The described infection studies of *Rousettus aegyptiacus* and *Eptesicus fuscus* with SARS-CoV-2 have only limited significance as CoV are described as strongly host specific. The SARS-like CoV in Europe were predominantly detected in *Rhinolophus hipposideros*, *R. ferrumequinum,* and *R. blasii*. To determine whether European bats are susceptible to SARS-CoV-2, European bats of the family *Rhinolophidae* would have to be investigated in further studies. In this proposed study it should also be investigated whether the viral loads excreted by a SARS-CoV-2-infected person were sufficient for an air-borne infection of the bats. For SARS-CoV-2 an average viral load in sputum of 7.00 × 10 × 6 copies per ml is reported [252]. Nevertheless, we should be aware and prevent a possible establishment of SARS-CoV-2 within the European bat populations. When viruses acquire new hosts (host jumps), it is often associated with a period of accelerated sequence change [253]. During this adaptation time the virus may remodel and regain fitness in the altered environment. Subsequently, this is typically associated with amino acid sequence changes of viral genes encoding receptor interactions and evasion of the innate immune system, but often throughout the entire virus genome [253,254,255,256]. On the one hand, the European *Rhinolophus* spp. are related to the Asian *Rhinolophidae* and host jumps may result in only lower evolutionary pressure. Phylogeographical reconstruction of the evolutionary history of the greater Horseshoe bat (*Rhinolophus ferrumequinum*) across Europe and west Asia revealed that nearly all of the European *Rhinolophus ferrumequinum* species were made up by a single haplotype spread from west Asia throughout Europe approximately 40,000–60,000 years ago [181]. On the other hand, it is hard to predict how these effects would either increase or decrease pathogenicity, virulence, and vaccine efficacy. However, successful establishment of SARS-CoV-2 within the European bat populations would provide a potential source of reintroducing the (altered) virus into the human population. 

As long as no further data are available to rule out a potential risk of anthropozoonotic transmission, it is good practice that bat volunteers and researchers wear FFP2 masks and gloves to prevent air-borne zoonotic and anthropozoonotic transmission, as is already recommended by most bat rehabilitation foundations (i.e., https://www.fledermausschutz.de/2020/12/29/fledermausschutz-empfehlungen-zur-kontrolle-von-winterquartieren-in-zeiten-von-corona/, accessed on 22 June 2021).

#### 3.1.3. Examining the Zoonotic Potential of Viruses in the Laboratory 

How can we continue to investigate the zoonotic potential, mostly starting with virus sequences revealed by virus discovery studies? There are several options to investigate viruses further. On the genomic side we can sequence the full genome, annotate proteins, calculate phylogenetic reconstructions and molecular clocks, analyze recombination and reassortment, and predict and compare genes and protein structures of interest (i.e., receptor-binding domains). All of these methods aim to find structures and genes related to human-pathogenic viruses. Virus isolation enables animal experiments, cell culture experiments, metatranscriptomics, and serostudies. Especially the availability of cell cultures of potential reservoirs is increasing which can be used for receptor studies and provide opportunities to examine species barriers. Proteomics, modeling, and many more techniques are more comprehensively available. Serosurveys in human and bat hosts are of importance, as they can give a retrospective picture of infection occurrence. However, the only indubitable proof of a zoonotic infection is the repeated isolation (persistence) of a virus from animal host and human. 

### 3.2. How Can We Assess the Zoonotic Risk?

Numerous general factors contribute to a potential risk of spillover, ranging from the abundancy of potential bat vectors to the innate immune response of the human hosts [233,257]. We have to collect the necessary data to be able to assess viral traits. Most virus discovery studies performed for European bats (and bats worldwide) describe new viral sequences and their phylogenic reconstruction. This is very important in order to be able to classify whether or not the newly discovered virus is potentially human pathogenic. With this data it can be decided which viruses have high priority for further investigation. If we want to draw conclusions on the zoonotic potential we need to go further and collect more data on virus–host dynamics. It is crucial to know whether the bats are shedding infectious virus particles or if they are just excreting non-infectious nucleic acids. It should be also considered that viral shedding may be subject to seasonal effects. With this data we could calculate the prevalence of the new viruses within the host population. Subsequently, we can set the data in context of ecological traits. Whether the bat species migrates over vast distances or roosts in human dwellings may affect any zoonotic potential. Plowright et al. describe exemplarily for Hendra virus that, for successful spillover, shedding must align with exposure behavior and susceptibility of the recipient hosts and with environmental and bat population conditions that generate levels of pathogen pressure that are sufficient to produce an infectious dose [257]. 

We have compared available data for those viruses which in our opinion may pose a potential threat to public health, based on their virological properties like relatedness to known human-pathogenic viruses (Table 4). We filled the Table with available data which should contribute to a risk assessment regarding a zoonotic potential. We considered the migratory behavior of bats as a potential risk for epizootic transmission and spread through diverse bat colonies. Assuming that immunity of the bat host follows recovery, viruses may disappear locally but persist globally through migration [258]. We have included the IUCN threat status. While examining global shifts of mammalian populations in the light of spillover risk, Johnson et al. found that species of least concern with increasing abundance were estimated to be 1.5 times the number of zoonotic viruses. Vulnerable species had less than one-sixth the number of viruses compared to species of least concern that were stable in abundance [4]. Synanthropic bat species are described to increase their abundance with the growing human population [259]. Synanthropic bat species may benefit from the energetic advantages of buildings (warmer roosts) to exploit habitats otherwise devoid of roosting structures [259]. Furthermore, the synanthropic nature of bat species is a requirement when thinking of bat–human contact as a prerequisite for spillover, beside bat handlers and tourists visiting bat caves. Bat Lyssavirus 1 (EBLV-1) was included as an example of a well-studied virus for which the necessary data are already available. 

Summarized, criteria used were (1) relatedness to a viral species known to induce severe diseases in humans; (2) viral RNA load shed by host species in copies/µL; (3) successful virus isolation; (4) infectious virus shedding; (5) potential route of transmission; (6) hints of epizootic or zoonotic transmission; (7) migration behavior of bat host; (8) IUCN threat; and (9) synanthropic behavior. These criteria were selected in accordance with the available literature [226,233,257,260]. Table 4 summarizes the research gaps we are currently facing for the newly discovered and potentially zoonotic viruses. Not all of these gaps can be closed easily nor is unlimited funding and manpower available. However, it is important to critically revise the available data, point out gaps, and propose to fill them.

## 4. Conclusions and Recommendations

Survey of European wildlife (especially bats) should be increased because the risk of zoonotic emerging diseases in Europe seems neglected. So far, several studies have enlightened the virome of European bats, many of which are comparable. However, research is also competitive in publishing the first sequences of certain viruses. Maybe it is time to overcome this because so much more could be achieved with a collaborative initiative. If bat researchers combined their skills and finalized a certain strategy it would become possible to address the missing gaps collaboratively. For example, a bat Filovirus (LLOV) was detected in *Miniopterus schreibersii* in Spain and Hungary. As most Filoviruses are described to be highly pathogenic for humans, the occurrence of LLOV should be carefully monitored. *Miniopterus* is a seasonally migrating species with flying distances between a few hundred and 800 km. There must be more *Miniopterus schreibersii* colonies in between Spain and Hungary that could serve as potential reservoirs of LLOV. It is assumed that the Spanish and French bats migrate from Africa through the Rhône valley and the Hungarian bats migrate over the eastern route through Turkey. However, the colonies of *Miniopterus schreibersii* have exchanges at a certain level. This would be a great opportunity to bundle ecological and virological expertise and skills throughout Europe to monitor and evaluate the occurrence of LLOV in *Miniopterus schreibersii*. Bat researchers of all countries participating could sample *Miniopterus schreibersii* colonies in their respective geographical research area. All samples could be investigated with the same coordinated methods, allowing to get a picture of LLOV prevalence in Europe. Furthermore, LLOV has been associated with mass mortality in *Miniopterus schreibersii*; raising awareness for this phenomenon across Europe could improve the timely investigation of LLOV emergence. This is just one example [28,69].

People are increasingly concerned about the risk posed by synanthropic bats (e.g., roosting in the attics of their houses). Viruses have been detected in numerous synanthropic species, therefore a potential for transmission is given (especially true for bat Lyssaviruses), though preventable by simple measures: No touching or handling of bats or bat excrements without gloved hands and, in the case of a bat bite, immediately proceeding to the appropriate facility for post-exposure prophylaxis [195]. Based on our current knowledge, zoonotic spillover events are extremely rare.

The intensified research effort on bat CoV after the emergence of SARS-CoV allowed for the rapid identification of SARS-CoV-2 and its potential reservoir host. This is an excellent example of the importance of knowing viruses harbored by bats for preparedness against emerging infectious diseases [85]. In most cases virus discovery studies are a snapshot of the viral diversity, and successful detection depends on several factors like seasonality, sample quality, ecological factors, and detection strategies. However, most of the viruses harbored by bats seem to be strictly species specific, and zoonotic events may be only very rare and unlikely. Only two viral genera proved to be zoonotic in Europe, the bat Lyssaviruses and the bat MRVs (see Section 1.2.5 and Section 1.2.6). However, also for Issyk-Kul virus strain PbGER recently discovered in Germany, a potential zoonotic transmission seems likely as Issyk-Kul virus has already been causing smaller endemics in Central Asia. For bat Lyssaviruses of phylogroup 1, the classical rabies virus vaccine confers cross-protection [104]. Bat MRV infection seems to be very rare and causes rather mild diseases [205,206]. However, even though there are only two proved zoonotic viruses, there are several viruses with zoonotic potential: at least all of the viruses in Table 4 should be subject to a thorough monitoring in Europe. In addition to the projected research studies filling the identified gaps in Table 4, seroprevalence studies should be conducted to estimate the prevalence of antibodies to bat viruses in the human population. Thorough longtime surveys on bats regarding seasonal viral shedding and generation of novel variants should be performed, alongside a comprehensive molecular surveillance system monitoring viruses beyond country borders in Europe. Another important consideration is the aspect of climate change and how it affects the European bat populations. By shifting populations to other European regions, the whole ecosystem will be affected. These effects are already being discussed as drivers of the SARS-CoV and SARS-CoV-2 pandemics in Asia [261]. The consequences for bat populations, viral dynamics, and shedding have to be carefully monitored.

## Figures and Tables

**Table 1:** Overview on virus data per bat species recorded at DBatVir [1].

	*Adenoviridae*	*Astroviridae*	*Bornaviridae*	*Caliciviridae*	*Circoviridae*	*Coronaviridae*	*Filoviridae*	*Flaviviridae*	*Hantaviridae*	*Hepeviridae*	*Herpesviridae*	*Nairoviridae*	*Papillomaviridae*	*Paramyxoviridae*	*Parvoviridae*	*Phenuiviridae*	*Picornaviridae*	*Polyomaviridae*	*Poxviridae*	*Reoviridae*	*Retroviridae*	*Rhabdoviridae*	*Total*
***Barbastella barbastellus***	0	1	0	0	0	0	0	0	0	0	0	0	0	0	0	0	0	0	0	0	0	0	1
***Eidolon helvum***	0	0	0	0	0	0	0	0	0	0	0	0	1	0	0	0	0	0	0	0	0	0	1
***Eptesicus isabellinus***	0	0	0	0	0	1	0	0	0	0	3	0	4	0	0	0	0	0	0	0	0	13	21
***Eptesicus nilssonii***	1	0	0	0	0	1	0	0	0	0	0	1	0	0	0	1	0	0	0	0	0	2	6
***Eptesicus serotinus***	3	1	0	1	0	2	0	0	0	1	3	0	13	0	0	0	0	0	0	2	1	315	342
***Hypsugo savii***	4	0	0	0	0	4	0	0	0	0	1	0	0	0	0	0	0	0	1	0	0	1	11
***Miniopterus schreibersii***	1	11	0	0	3	16	2	0	0	0	5	0	0	0	4	0	18	0	0	1	0	5	66
***Murina leucogaster***	0	0	0	0	0	0	0	0	0	0	0	0	0	0	0	0	0	0	0	0	0	1	1
***Myotis alcathoe***	0	0	0	1	1	0	0	0	0	0	1	0	0	1	0	0	0	0	0	0	0	0	4
***Myotis bechsteinii***	1	1	0	0	0	2	0	0	0	1	1	0	0	1	0	0	1	0	0	0	0	0	8
***Myotis blythii***	1	0	0	0	0	2	0	0	0	0	1	0	0	0	0	0	0	0	0	0	0	0	4
***Myotis brandtii***	0	0	0	0	0	2	0	0	0	0	0	0	0	0	0	0	0	0	0	0	0	1	3
***Myotis capaccinii***	0	3	0	0	0	6	0	0	0	0	2	0	0	1	0	0	0	0	0	0	0	0	12
***Myotis dasycneme***	2	0	0	0	0	23	0	0	0	0	0	0	0	0	0	0	2	0	0	0	0	9	36
***Myotis daubentonii***	0	1	0	1	0	63	0	0	0	2	2	0	0	8	0	0	0	0	0	8	0	47	132
***Myotis emarginatus***	4	3	0	0	1	26	0	0	0	0	2	0	0	9	0	0	0	0	0	1	0	0	46
***Myotis escalerai***	0	0	0	0	0	0	0	0	0	0	2	0	0	0	0	0	0	0	0	0	0	0	2
***Myotis myotis***	3	8	0	0	1	22	0	0	0	0	2	0	0	2	0	0	15	0	0	1	0	21	75
***Myotis myotis blythii***	0	1	0	0	0	0	0	0	0	0	0	0	0	0	0	0	0	0	0	0	0	0	1
***Myotis mystacinus***	1	1	0	0	0	0	0	0	0	0	3	1	0	4	0	0	0	0	0	3	0	1	14
***Myotis nattereri***	0	1	1	0	1	24	0	0	0	0	2	0	0	4	0	0	0	0	0	1	0	7	41
***Myotis oxygnathus***	0	0	0	0	0	1	0	0	0	0	0	0	0	0	0	0	1	0	0	0	0	0	2
***Nyctalus lasiopterus***	16	0	0	0	0	5	0	0	0	0	3	0	0	0	0	0	0	0	0	0	0	0	24
***Nyctalus leisleri***	8	0	0	0	0	1	0	0	0	0	2	0	0	0	0	0	0	0	0	0	0	0	11
***Nyctalus noctula***	13	2	0	0	1	5	0	0	2	0	3	0	0	1	1	0	2	0	0	0	0	0	30
***Pipistrellus***	0	1	0	0	0	3	0	0	0	0	0	0	0	0	0	0	0	0	0	0	0	0	4
***Pipistrellus kuhlii***	18	2	0	0	0	16	0	0	0	0	1	0	0	2	1	1	1	0	0	28	0	1	71
***Pipistrellus nathusii***	5	0	0	0	1	5	0	0	0	1	1	0	0	0	3	0	0	0	0	0	0	1	17
***Pipistrellus pipistrellus***	13	1	1	0	0	13	0	2	0	0	3	0	0	2	2	0	1	0	0	2	0	1	41
***Pipistrellus pygmaeus***	32	1	0	0	0	12	0	0	0	0	1	0	0	0	0	0	0	0	0	0	0	0	46
***Plecotus auritus***	1	1	0	0	1	1	0	0	0	0	1	0	0	0	2	0	0	0	0	1	0	3	11
***Plecotus austriacus***	2	0	0	0	0	0	0	0	0	0	2	0	0	0	0	0	0	0	0	0	0	0	4
***Pteropus giganteus***	0	0	0	0	0	0	0	0	0	0	0	0	1	0	0	0	0	0	0	0	0	0	1
***Pteropus vampyrus***	0	0	0	0	0	0	0	0	0	0	0	0	0	0	0	0	0	0	0	1	0	0	1
***Rhinolophus***	0	0	0	0	0	0	0	0	0	0	0	0	0	0	0	0	0	0	0	1	0	0	1
***Rhinolophus blasii***	0	0	0	0	0	10	0	0	0	0	0	0	0	0	0	0	1	0	0	2	0	0	13
***Rhinolophus euryale***	11	0	0	0	0	8	0	0	0	0	0	0	0	0	0	0	5	1	0	3	0	0	28
***Rhinolophus ferrumequinum***	11	0	0	0	0	99	0	0	0	0	1	0	1	0	0	0	3	0	0	1	1	2	119
***Rhinolophus hipposideros***	1	1	0	0	1	7	0	0	0	0	1	0	0	0	0	0	1	1	0	2	0	0	15
***Rhinolophus mehelyi***	0	0	0	0	0	2	0	0	0	0	0	0	0	0	0	0	0	0	0	0	0	0	2
***Rousettus aegyptiacus***	0	0	0	0	0	0	0	0	0	0	3	0	0	0	0	0	0	0	0	0	0	1	4
***Tadarida teniotis***	0	0	0	0	0	0	0	0	0	0	4	0	0	0	0	0	0	0	0	2	0	0	6
***Vespertilio murinus***	1	1	0	0	1	0	0	0	0	0	0	0	0	0	0	0	0	0	0	2	0	3	8
**unclassified *Chiroptera***	0	0	0	0	0	3	0	0	0	0	0	2	0	0	0	1	1	0	0	3	0	56	66
**Total**	153	42	2	3	12	385	2	2	2	5	56	4	20	35	13	3	52	2	1	65	2	491	1352

**Table 2:** Overview on viruses detected in European bats with references (Data from DBatVir [1]).

Virus Family	Genus	Bat Species	Origin	Detection	Reference
*Adenoviridae*	Mastadenovirus	***Pipistrellus nathusii*** ***Pipistrellus pipistrellus***	**Germany**	**Isolation** **PCR**	[34,35]
		***Nyctalus noctule*** ***Rhinolophus ferrumequinum***	**Hungary**	**PCR**	[36]
		***Rhinolophus euryale*** ***Rhinolophus ferrumequinum*** ***Rhinolophus hipposideros*** ***Eptesicus nilssonii*** ***Eptesicus serotinus*** ***Myotis blythii*** ***Myotis dasycneme*** ***Myotis emarginatus*** ***Myotis myotis*** ***Myotis mystacinus*** ***Nyctalus leisleri*** ***Nyctalus noctula*** ***Pipistrellus kuhlii*** ***Pipistrellus nathusii*** ***Pipistrellus pipistrellus*** ***Pipistrellus pygmaeus*** ***Plecotus auratus*** ***Vespertilio murinus***	**Hungary/Germany**	**PCR**	[37]
		***Myotis myotis***	**Germany**	**PCR**	[38]
		***Hypsugo savii*** ***Myotis bechsteinii*** ***Myotis emarginatus*** ***Myotis myotis*** ***Nyctalus noctula*** ***Nyctalus lasiopterus*** ***Nyctalus leisleri*** ***Pipistrellus kuhlii*** ***Pipistrellus pipistrellus*** ***Pipistrellus pygmaeus*** ***Rhinolophus euryale*** ***Rhinolophus ferrumequinum***	**Spain**	**PCR**	[39]
		***Pipistrellus kuhlii***	**Italy**	**Isolation**	[40]
*Astroviridae*	Mamastrovirus	***Myotis*** ***myotis***	**Germany**	**PCR**	[38]
		***Myotis daubentonii*** ***Plecotus auritus*** ***Myotis bechsteinii*** ***Nyctalus noctula*** ***Pipistrellus pygmaeus*** ***Myotis emarginatus*** ***Myotis nattereri*** ***Miniopterus schreibersii***	**Hungary**	**PCR**	[41,42]
		***Pipistrellus*** **spp.** ***Myotis mystacinus*** ***Myotis emarginatus*** ***Pipistrellus pipistrellus*** ***Vespertilio murinus*** ***Nyctalus noctule*** ***Rhinolophus hipposideros***	**Czech Republic**	**PCR**	[43]
		***Barbastella barbastellus*** ***Eptesicus serotinus*** ***Miniopterus schreibersii*** ***Myotis capaccinii*** ***Myotis emarginatus*** ***Myotis myotis blythii*** ***Pipistrellus kuhlii***	**Italy**	**PCR**	[44]
*Bornaviridae*		***Myotis nattereri*** ***Pipistrellus pipistrellus***	**France**	**Metagenomics**	[22]
*Caliciviridae*		***Eptesicus serotinus*** ***Myotis alcathoe*** ***Myotis daubentonii***	**Hungary**	**PCR**	[42]
*Circoviridae*		***Miniopterus schreibersii***	**Italy**	**PCR**	[45]
		***Miniopterus schreibersii***	**Croatia**	**Metagenomics**	[46]
		***Myotis nattereri*** ***Myotis emarginatus*** ***Myotis alcathoe*** ***Plecotus auritus*** ***Pipistrellus nathusii*** ***Nyctalus noctula***	**Hungary, Serbia, Ukraine**	**Metagenomics**	[24]
*Bunyaviridae*	Phenuivirus	***Pipistrellus*** ***kuhlii***	**Italy**	**Isolation**	[47]
		***Eptesicus nilssonii***	**Germany**	**Metagenomics**	[26]
	Nairovirus	***Myotis mystacinus***	**France**	**Metagenomics**	[22]
		***Eptesicus nilssonii***	**Germany**	**Metagenomics**	[15,25]
*Coronaviridae*	Alphacoronavirus	***Myotis bechsteinii*** ***Myotis dasycneme*** ***Myotis daubentonii*** ***Pipistrellus nathusii*** ***Pipistrellus pygmaeus*** ***Myotis nattereri***	**Germany**	**PCR**	[48,49]
		***Pipistrellus pipistrellus***	**Germany**	**Metagenomics**	[15]
		***Myotis blythii*** ***Myotis daubentonii*** ***Myotis myotis*** ***Mineropterus schreibersii*** ***Nyctalus lasiopterus*** ***Pipistrellus kuhlii*** ***Pipistrellus*** **spp.**	**Spain**	**PCR**	[50]
		***Rhinolophus ferrumequinum*** ***Myotis emarginatus*** ***Myotis daubentonii*** ***Myotis nattereri*** ***Rhinolophus ferrumequinum*** ***Myotis myotis*** ***Miniopterus schreibersii*** ***Myotis capaccinii*** ***Pipistrellus pipistrellus***	**France, Spain**	**PCR**	[51,52]
		***Myotis brandtii*** ***Myotis daubentoniid*** ***Eptesicus nilssonii***	**Finland**	**PCR**	[53]
		***Myotis myotis*** ***Myotis nattereri*** ***Myotis oxygnathus*** ***Plecotus auritus*** ***Pipistrellus kuhlii*** ***Pipistrellus pipistrellus*** ***Rhinolophus ferrumequinum***	**Italy**	**PCR**	[54,55,56,57]
		***Hypsugo savii*** ***Nyctalus noctule*** ***Pipistrellus kuhlii*** ***Pipistrellus*** **spp.** ***Rhinolophus hipposideros***	**Italy**	**PCR**	[57]
		***Miniopterus schreibersii*** ***Nyctalus leisleri*** ***Rhinolophus euryale*** ***Rhinolophus blasii*** ***Rhinolophus ferrumequinum*** ***Rhinolophus mehelyi***	**Germany**	**PCR**	[38]
		***Myotis daubentoniid*** ***Myotis nattereri***	**United Kingdom**	**PCR**	[58]
		***Myotis daubentonii*** ***Myotis dasycneme*** ***Eptesicus serotinus*** ***Pipistrellus pygmaeus*** ***Myotis nattereri***	**Denmark**	**PCR**	[59]
		***Myotis myotis*** ***Pipistrellus pygmaeus*** ***Myotis nattereri*** ***Rhinolophus ferrumequinum*** ***Rhinolophus hipposideros*** ***Myotis daubentonii***	**Hungary**	**PCR**	[42]
		***Myotis emarginatus*** ***Rhinolophus ferrumequinum***	**Luxembourg**	**PCR**	[60]
	Betacoronavirus	***Miniopterus schreibersii*** ***Nyctalus leisleri*** ***Myotis daubentonii*** ***Rhinolophus euryale*** ***Rhinolophus blasii*** ***Rhinolophus ferrumequinum*** ***Rhinolophus mehelyi*** ***Rhinolophus*** ***hipposideros***	**Bulgaria** **Germany**	**PCR**	[61]
		***Myotis brandtii*** ***Eptesicus nilssonii***	**Finland**	**PCR**	[53]
		***Rhinolophus euryale***	**Hungary**	**PCR**	[42,62]
		***Rhinolophus ferrumequinum***	**Luxembourg**	**PCR**	[60]
		***Pipistrellus nathusii*** ***Pipistrellus pygmaeus*** ***Pipistrellus pipistrellus***	**Romania, Ukraine**	**PCR**	[63]
		***Rhinolophus hipposideros***	**Slovenia**	**PCR**	[64]
		***Pipistrellus pipistrellus***	**Netherlands**	**PCR**	[65]
		***Rhinolophus hipposideros***	**United Kingdom**	**Metagenomics**	[27] Preprint
		***Eptesicus isabellinus*** ***Hypsugo savii***	**Spain**	**PCR**	[50]
		***Eptesicus serotinus*** ***Hypsugo savii*** ***Nyctalus noctule*** ***Pipistrellus kuhlii*** ***Pipistrellus* sp.** ***Rhinolophus hipposideros*** ***Rhinolophus ferrumequinum***	**Italy**	**PCR**	[54,56,57,66,67,68]
		***Rhinolophus ferrumequinum***	**France, Spain**	**PCR**	[51]
*Filoviridae*	Cuevavirus	***Miniopterus*** ***schreibersii***	**Spain, Hungary**	**PCR**	[28,69]
*Flaviviridae*	Japanese encephalitis serocomplex	***Pipistrellus pipistrellus***	**Germany**	**PCR**	[70]
*Hantavirus*		***Nyctalus noctula***	**Czech Republic**	**PCR**	[71]
*Hepeviruses*	Hep-E-related viruses	***Eptesicus serotinus*** ***Myotis bechsteinii*** ***Myotis daubentonii***	**Germany** **Bulgaria**	**PCR**	[72]
*Herpesviridae*	Betaherpesvirus Gammaherpesvirus	***Myotis myotis*** ***Myotis nattereri*** ***Nyctalus noctula*** ***Pipistrellus pipistrellus*** ***Plecotus auritus***	**Germany**	**PCR**	[73]
	Betaherpesvirus Alphaherpesviruses	***Rousettus*** ***aegyptiacus***	**Hungary**	**PCR**	[36]
	Betaherpesviruses	***Eptesicus isabellinus*** ***Hypsugo savii*** ***Miniopterus schreibersii*** ***Myotis alcathoe*** ***Myotis bechsteinii*** ***Myotis blythii*** ***Myotis capaccinii*** ***Myotis daubentonii*** ***Myotis emarginatus*** ***Myotis escalerai*** ***Myotis myotis*** ***Myotis mystacinus*** ***Myotis nattereri*** ***Nyctalus lasiopterus*** ***Nyctalus leisleri*** ***Nyctalus noctula*** ***Pipistrellus pipistrellus*** ***Pipistrellus kuhlii*** ***Pipistrellus pygmaeus*** ***Plecotus austriacus*** ***Rhinolophus ferrumequinum*** ***Rhinolophus hipposideros*** ***Rousettus aegyptiacus*** ***Tadarida teniotis***	**Spain**	**PCR**	[74]
	Gammaherpesvirus	***Eptesicus serotinus***	**Hungary**	**PCR**	[75]
*Papillomavirus*	Papillomavirus	***Eptesicus serotinus*** ***Rhinolophus ferrumequinum***	**Spain**	**PCR**	[76]
*Paramyxoviridae*	Unassigned	***Myotis mystacinus*** ***Nyctalus noctula*** ***Pipistrellus pipistrellus***	**Germany**	**PCR**	[77]
	Morbillivirus	***Myotis bechsteinii*** ***Myotis daubentonii*** ***Myotis myotis*** ***Myotis mystacinus*** ***Myotis alcathoe*** ***Myotis capaccinii***	**Bulgaria** **Germany** **Romania**	**PCR**	[78]
*Parvoviridae*		***Miniopterus schreibersii***	**Croatia**	**Metagenomics**	[23]
		***Miniopterus schreibersii***	**Hungary**	**Metagenomics**	[29]
		***Myotis myotis*** ***Pipistrellus kuhlii*** ***Myotis nattereri*** ***Eptesicus nilssonii*** ***Myotis daubentoniid*** ***Vespertilio murinus*** ***Eptesicus nilssonii*** ***Nyctalus noctula***	**Germany**	**Metagenomics**	[15]
*Picornaviridae*		***Rhinolophus ferrumequinum*** ***Myotis myotis*** ***Pipistrellus kuhlii*** ***Nyctalus noctula*** ***Rhinolophus hipposideros*** ***Miniopterus schreibersii*** ***Myotis dasycneme***	**Luxembourg, Germany, Spain, Romania**	**PCR**	[79]
		***Miniopterous schreibersii***	**Hungary**	**Metagenomics**	[31]
		***Pipistrellus pipistrellus***	**Italy**	**Metagenomics**	[30]
		***Plecotus aurithus*** ***Pipistrellus nathusii***	**Germany**	**Metagenomics**	[15]
*Polyomavirus*		***Rhinolophus euryale*** ***Rhinolophus hipposideros***	**Hungary**	**PCR**	[80]
*Poxviridae*		***Hypsugo savii***	**Italy**	**Isolation**	[81]
*Reoviridae*	Orthoreovirus	***Myotis mystacinus*** ***Nyctalus noctula*** ***Pipistrellus pipistrellus*** ***Pipistrellus nathusii*** ***Pipistrellus kuhlii*** ***Plecotus auritus***	**Germany**	**Isolation** **PCR**	[82]
		***Pipistrellus kuhlii*** ***Rhinolophus hipposideros*** ***Nyctalus noctula*** ***Tadarida teniotis*** ***Nyctalus noctula***	**Italy**	**Isolation** **PCR**	[83]
		***Myotis nattereri*** ***Pipistrellus kuhlii***	**Italy**	**PCR**	[33]
		***Eptesicus serotinus*** ***Myotis daubentonii*** ***Myotis myotis*** ***Myotis emarginatus***	**Slovenia**	**PCR**	[84]
	Rotavirus	***Rhinolophus blasii*** ***Rhinolophus*** ***Rhinolophus euryale*** ***Myotis daubentonii***	**Germany,** **Bulgaria**	**PCR**	[85]
		***Myotis mystacinus***	**France**	**Metagenomics**	[22]
		***Pipistrellus pipistrellus***	**Germany**	**Metagenomics**	[15]
		***Miniopterus schreibersii***	**Serbia**	**Metagenomics**	[32]
	Orbivirus	***Nyctalus noctula***	**Germany**	**Metagenomics**	[15]
*Retroviridae*	Gammaretrovirus	***Eptesicus serotinus***	**France**	**Metagenomics**	[22]
	Endogenous Retrovirus	*Myotis myotis* *Pipistrellus kuhlii* *Pipistrellus pipistrellus* *Myotis daubentoniid* *Vespertilio murinus*	Germany	**Metagenomics**	[15]
*Rhabdoviridae*	Various European bat lyssaviruses	*Eptesicus serotinus* *Eptesicus isabellinus* *Hypsugo savii* *Miniopterus schreibersii* *Myotis myotis* *Myotis daubentonii* *Myotis dasycneme* *Myotis nattereri* *Myotis brandtii* *Plectorus auritus* *Pipistrellus pipistrellus* *Pipistrellus kuhlii* *Rhinolophus ferrumequinum* *Rousettus aegyptiacus* *Vespertilio murinus* unclassified *Chiroptera*	Denmark France Finland Germany Hungary Italy Netherlands Norway Poland Slovakia Spain Switzerland Ukraine United Kingdom	**Microscopy** **Isolation** **PCR**	[86,87,88,89,90,91,92,93,94,95,96,97,98,99,100,101,102,103,104,105,106,107,108,109] [92,93,94,95,101,108,109,110,111]

**Table 3:** Migrating bat species in Europe (sedentary species (up to 100 km of movement), seasonally migrating species (up to 800 km) and long-distance migrants (up to 4000 km)) [219].

Sedentary Species	Seasonal Migrants	Long-Distance Migrants
*Rhinolophus blasii*, *R. euryale*, *R. ferrumequinum*, *R. hipposideros*, *R. mehelyi*, *Myotis bechsteinii*, *M. emarginatus*, *M. nattereri*, *Pipistrellus kuhlii*, *Plecotus auritus*, *P. austriacus*, *P. teneriffae*, *Tadarida teniotis*	*Barbastella barbastellus*, *Eptesicus nilssonii*, *E. serotinus*, *Myotis blythii*, *M. brandtii*, *M. capaccinii*, *M. dasycneme*, *M.daubentonii*, *M. myotis*, *M. mystacinus*, *Pipistrellus pipistrellus*, *Miniopterus schreibersii*	*Nyctalus leisleri*, *Nyctalus noctula*, *Pipistrellus nathusii*, *Vespertilio murinus*

**Table 4:** Overview on risk factors that may contribute to zoonotic transmission and spillover. Bat species: E. Ser, Eptesicus serotinus; R. hip, Rhinolophus hipposideros; R. fer, Rhinolophus ferrumequinum; R. bla, Rhinolophus blasii; M. sch, Miniopterous schreibersii; P. pip, Pipistrellus pipistrellus; E. nil, Eptesicus nilssonii; N. noc, Nyctalus noctula; P. aur, Plecotus auritus. Migration: seasonal; seasonal migrants; long distance; long-distance migrants. Human interaction: syn, synanthropic species; synNE, synanthropic in northern Europe; non-syn, non-synanthropic. * copies per gram of feces.

Virus Name	Country Bat Species	Related to Viral Family/Order	Viral RNA (copies/µL)	Virus Isolated	Virus Shedding	Potential Shedding Route	Hints for Epizootic or Zoonotic Transmission	Migration	IUCN	Human Interaction
**EBLV-1**	Europe *E. ser* EpE.	*Rhabdoviridae*	CT > 20 (salivary glands)	Yes	no data	oral, bites	zoonotic	seasonal	least concern	syn
**BtCoV 187632-2/2012**	Italy *R. hip*	*Coronaviridae* Sarbecovirus	no data	Neg	no data	fecal	no data	sedentary	least concern	synNE
**BtCoV 243585/2012**	Italy *R. hip*	*Coronaviridae* Sarbecovirus	no data	Neg	no data	fecal	no data	sedentary	least concern	synNE
**BtCoV 19681/2011**	Italy *R. hip*	*Coronaviridae* Sarbecovirus	no data	Neg	no data	fecal	no data	sedentary	least concern	synNE
**SarBatCoV1**	Italy *R. fer*	*Coronaviridae* Sarbecovirus	no data	no data	no data	fecal	no data	sedentary	least concern	synNE
**BtCoV 893/09-11**	Italy *R. fer*	*Coronaviridae* Sarbecovirus	no data	no data	no data	fecal	no data	sedentary	least concern	synNE
**SLO1A00XX**	Slovenia *R. hip*	*Coronaviridae* Sarbecovirus	no data	CoV particle (EM)	no data	fecal	no data	sedentary	least concern	synNE
**BtCoV FRA_EPI1_3975**	France *R. fer*	*Coronaviridae* Sarbecovirus	no data	no data	no data	fecal	no data	sedentary	least concern	synNE
**BtCoV LUX16_A_2016**	Luxembourg *R. fer*	*Coronaviridae* Sarbecovirus	no data	no data	no data	fecal	no data	sedentary	least concern	synNE
**BtCoV BM48-31/BGR/2008**	Bulgaria *R. bla*	*Coronaviridae* Sarbecovirus	2.4 × 10^8^ *	Neg	no data	fecal	no data	seasonal mig	vulnerable	synNE
**Lloviu virus**	Spain, Hungary *M. sch*	*Filoviridae* Cuevavirus	1.6 × 10^4^	Neg	no data	fecal + aerosol *	no data	seasonal	least concern	non-syn
**Usutu virus**	Germany *P. pip*	*Flaviviridae* JEV complex	no data	Neg	no data	? (brain)	epizootic	seasonal	least concern	syn
**Issyk-Kul virus PbGER**	Germany *E. nil*	*Nairoviridae* Keterah	3.5 × 10^6^ (liver), 7.6 × 10^4^ (lungs)	Neg	no data	aerosol *	zoonotic	seasonal	least concern	syn
**Zwiesel bat banyangvirus**	Germany *E. nil*	*Nairoviridae* Banyangvirus	4.0 × 10^6^ (spleen)	Neg	no data	? (liver, lungs, spleen, intestine)	no data	seasonal	least concern	syn
**Brno virus**	Czech Republic *N. noc*	Bat-associated Hantavirus	no data	Neg	no data	? (liver, kidney)	no data	long distance	least concern	syn
**T3/Bat/Germany/342/08**	Germany *P. aur*	Mammalian orthoreovirus	2.4 × 10^7^ (intestine)	Yes	no data	fecal	epizootic, zoonotic	sedentary	least concern	syn
**SI-MRV0/SI-MRV02**	Slovenia *E. ser*	Mammalian orthoreovirus	no data	Yes	no data	fecal	zoonotic	seasonal	least concern	syn

## References

[1] Chen L., Liu B., Yang J., Jin Q. (2014). DBatVir: The database of bat-associated viruses. Database.

[2] Kohl C., Kurth A. (2014). European bats as carriers of viruses with zoonotic potential. Viruses.

[3] Cisneros L.M., Burgio K.R., Dreiss L.M., Klingbeil B.T., Patterson B.D., Presley S.J., Willig M.R. (2014). Multiple dimensions of bat biodiversity along an extensive tropical elevational gradient. J. Anim. Ecol..

[4] Johnson C.K., Hitchens P.L., Pandit P.S., Rushmore J., Evans T.S., Young C.C.W., Doyle M.M. (2020). Global shifts in mammalian population trends reveal key predictors of virus spillover risk. Proc. Biol. Sci..

[5] Kissi B., Tordo N., Bourhy H. (1995). Genetic polymorphism in the rabies virus nucleoprotein gene. Virology.

[6] Halpin K., Young P., Field H., Mackenzie J. (2000). Isolation of Hendra virus from pteropid bats: A natural reservoir of Hendra virus. J. Gen. Virol..

[7] Yob J.M., Field H., Rashdi A.M., Morrissy C., van der Heide B., Rota P., bin Adzhar A., White J., Daniels P., Jamaluddin A. (2001). Nipah Virus Infection in Bats (Order Chiroptera) in Peninsular Malaysia. Emerg. Infect. Dis..

[8] Li W., Shi Z., Yu M., Ren W., Smith C., Epstein J.H., Wang H., Crameri G., Hu Z., Zhang H. (2005). Bats are natural reservoirs of SARS-like coronaviruses. Science.

[9] Towner J.S., Amman B.R., Sealy T.K., Carroll S.A.R., Comer J.A., Kemp A., Swanepoel R., Paddock C.D., Balinandi S., Khristova M.L. (2009). Isolation of Genetically Diverse Marburg Viruses from Egyptian Fruit Bats. PLoS Pathog..

[10] Leroy E.M., Kumulungui B., Pourrut X., Rouquet P., Hassanin A., Yaba P., Délicat A., Paweska J.T., Gonzalez J.P., Swanepoel R. (2005). Fruit bats as reservoirs of Ebola virus. Nature.

[11] Memish Z.A., Mishra N., Olival K.J., Fagbo S.F., Kapoor V., Epstein J.H., AlHakeem R., Al Asmari M., Islam A., Kapoor A. (2013). Middle East respiratory syndrome coronavirus in Bats, Saudi Arabia. Emerg. Infect. Dis..

[12] Zhou P., Yang X.L., Wang X.G., Hu B., Zhang L., Zhang W., Si H.R., Zhu Y., Li B., Huang C.L. (2020). A pneumonia outbreak associated with a new coronavirus of probable bat origin. Nature.

[13] Cirkovic V., Stamenkovic G., Jovanovic J., Siljic M., Paunovic M., Stanojevic M. (2016). Failure to detect viral RNA in bat samples collected in the Balkan region. Trop. Biomed..

[14] Fereidouni S., Kwasnitschka L., Balkema Buschmann A., Muller T., Freuling C., Schatz J., Pikula J., Bandouchova H., Hoffmann R., Ohlendorf B. (2015). No virological evidence for an influenza A-like virus in European bats. Zoonoses Public Health.

[15] Kohl C., Brinkmann A., Radonic A., Dabrowski P.W., Muhldorfer K., Nitsche A., Wibbelt G., Kurth A. (2021). The virome of German bats: Comparing virus discovery approaches. Sci. Rep..

[16] Nobach D., Herden C. (2020). No evidence for European bats serving as reservoir for Borna disease virus 1 or other known mammalian orthobornaviruses. Virol. J..

[17] Muhldorfer K., Speck S., Kurth A., Lesnik R., Freuling C., Muller T., Kramer-Schadt S., Wibbelt G. (2011). Diseases and causes of death in European bats: Dynamics in disease susceptibility and infection rates. PLoS ONE.

[18] Canard B., Sarfati R.S. (1994). DNA polymerase fluorescent substrates with reversible 3′-tags. Gene.

[19] Margulies M., Egholm M., Altman W.E., Attiya S., Bader J.S., Bemben L.A., Berka J., Braverman M.S., Chen Y.J., Chen Z. (2005). Genome sequencing in microfabricated high-density picolitre reactors. Nature.

[20] Mikheyev A.S., Tin M.M.Y. (2014). A first look at the Oxford Nanopore MinION sequencer. Mol. Ecol. Resour..

[21] Brinkmann A., Ergunay K., Radonic A., Kocak Tufan Z., Domingo C., Nitsche A. (2017). Development and preliminary evaluation of a multiplexed amplification and next generation sequencing method for viral hemorrhagic fever diagnostics. PLoS Neglect. Trop. Dis..

[22] Dacheux L., Cervantes-Gonzalez M., Guigon G., Thiberge J.M., Vandenbogaert M., Maufrais C., Caro V., Bourhy H. (2014). A preliminary study of viral metagenomics of French bat species in contact with humans: Identification of new mammalian viruses. PLoS ONE.

[23] Simic I., Zorec T.M., Lojkic I., Kresic N., Poljak M., Cliquet F., Picard-Meyer E., Wasniewski M., Zrncic V., Cukusic A. (2020). Viral Metagenomic Profiling of Croatian Bat Population Reveals Sample and Habitat Dependent Diversity. Viruses.

[24] Kemenesi G., Kurucz K., Zana B., Foldes F., Urban P., Vlaschenko A., Kravchenko K., Budinski I., Szodoray-Paradi F., Bucs S. (2018). Diverse replication-associated protein encoding circular DNA viruses in guano samples of Central-Eastern European bats. Arch. Virol..

[25] Brinkmann A., Kohl C., Radonic A., Dabrowski P.W., Muhldorfer K., Nitsche A., Wibbelt G., Kurth A. (2020). First detection of bat-borne Issyk-Kul virus in Europe. Sci. Rep..

[26] Kohl C., Brinkmann A., Radonic A., Dabrowski P.W., Nitsche A., Muhldorfer K., Wibbelt G., Kurth A. (2020). Zwiesel bat banyangvirus, a potentially zoonotic Huaiyangshan banyangvirus (Formerly known as SFTS)-like banyangvirus in Northern bats from Germany. Sci. Rep..

[27] Crook J., Murphy I., Carter D., Pullan S., Carroll M., Vipond R., Cunningham A., Bell D. (2021). Metagenomic Identification of a New Sarbecovirus from Horseshoe Bats in Europe. Sci. Rep..

[28] Kemenesi G., Kurucz K., Dallos B., Zana B., Foldes F., Boldogh S., Gorfol T., Carroll M.W., Jakab F. (2018). Re-emergence of Lloviu virus in Miniopterus schreibersii bats, Hungary, 2016. Emerg. Microbes Infect..

[29] Kemenesi G., Dallos B., Gorfol T., Estok P., Boldogh S., Kurucz K., Oldal M., Marton S., Banyai K., Jakab F. (2015). Genetic diversity and recombination within bufaviruses: Detection of a novel strain in Hungarian bats. Infect. Genet. Evol..

[30] Diakoudi G., Jamnikar-Ciglenecki U., Lanave G., Lelli D., Martella V., Kuhar U. (2020). Genome sequence of an aichivirus detected in a common pipistrelle bat (*Pipistrellus pipistrellus*). Arch. Virol..

[31] Kemenesi G., Zhang D., Marton S., Dallos B., Gorfol T., Estok P., Boldogh S., Kurucz K., Oldal M., Kutas A. (2015). Genetic characterization of a novel picornavirus detected in *Miniopterus schreibersii* bats. J. Gen. Virol..

[32] Banyai K., Kemenesi G., Budinski I., Foldes F., Zana B., Marton S., Varga-Kugler R., Oldal M., Kurucz K., Jakab F. (2017). Candidate new rotavirus species in Schreiber’s bats, Serbia. Infect. Genet. Evol..

[33] Leopardi S., Priori P., Zecchin B., Zamperin G., Milani A., Tonon F., Giorgiutti M., Beato M.S., De Benedictis P. (2020). Interface between Bats and Pigs in Heavy Pig Production. Viruses.

[34] Sonntag M., Muhldorfer K., Speck S., Wibbelt G., Kurth A. (2009). New adenovirus in bats, Germany. Emerg. Infect. Dis..

[35] Kohl C., Vidovszky M.Z., Muhldorfer K., Dabrowski P.W., Radonic A., Nitsche A., Wibbelt G., Kurth A., Harrach B. (2012). Genome analysis of bat adenovirus 2: Indications of interspecies transmission. J. Virol..

[36] Janoska M., Vidovszky M., Molnar V., Liptovszky M., Harrach B., Benko M. (2011). Novel adenoviruses and herpesviruses detected in bats. Vet. J..

[37] Vidovszky M., Kohl C., Boldogh S., Gorfol T., Wibbelt G., Kurth A., Harrach B. (2015). Random sampling of the Central European bat fauna reveals the existence of numerous hitherto unknown adenoviruses. Acta Vet. Hung..

[38] Drexler J.F., Corman V.M., Wegner T., Tateno A.F., Zerbinati R.M., Gloza-Rausch F., Seebens A., Müller M.A., Drosten C. (2011). Amplification of Emerging Viruses in a Bat Colony. Emerg. Infect. Dis..

[39] Iglesias-Caballero M., Juste J., Vazquez-Moron S., Falcon A., Aznar-Lopez C., Ibanez C., Pozo F., Ruiz G., Berciano J.M., Garin I. (2018). New Adenovirus Groups in Western Palaearctic Bats. Viruses.

[40] Diakoudi G., Lanave G., Moreno A., Chiapponi C., Sozzi E., Prosperi A., Larocca V., Losurdo M., Decaro N., Martella V. (2019). Surveillance for Adenoviruses in Bats in Italy. Viruses.

[41] Kemenesi G., Dallos B., Gorfol T., Boldogh S., Estok P., Kurucz K., Oldal M., Nemeth V., Madai M., Banyai K. (2014). Novel European lineages of bat astroviruses identified in Hungary. Acta Virol..

[42] Kemenesi G., Dallos B., Gorfol T., Boldogh S., Estok P., Kurucz K., Kutas A., Foldes F., Oldal M., Nemeth V. (2014). Molecular survey of RNA viruses in Hungarian bats: Discovering novel astroviruses, coronaviruses, and caliciviruses. Vector Borne Zoonotic Dis..

[43] Dufkova L., Strakova P., Sirmarova J., Salat J., Moutelikova R., Chrudimsky T., Bartonicka T., Nowotny N., Ruzek D. (2015). Detection of Diverse Novel Bat Astrovirus Sequences in the Czech Republic. Vector Borne Zoonotic Dis..

[44] Amoroso M.G., Russo D., Lanave G., Cistrone L., Pratelli A., Martella V., Galiero G., Decaro N., Fusco G. (2018). Detection and phylogenetic characterization of astroviruses in insectivorous bats from Central-Southern Italy. Zoonoses Public Health.

[45] Lecis R., Mucedda M., Pidinchedda E., Zobba R., Pittau M., Alberti A. (2020). Genomic characterization of a novel bat-associated Circovirus detected in European Miniopterus schreibersii bats. Virus Genes.

[46] Simic I., Zorec T.M., Kresic N., Poljak M., Bedekovic T., Lojkic I. (2019). Novel Circo-Like Virus Detected in a Croatian Bat Population. Microbiol. Resour. Announc..

[47] Baggieri M., Marchi A., Bucci P., Nicoletti L., Magurano F. (2015). Genetic variability of the S segment of Toscana virus. Virus Res..

[48] Gloza-Rausch F., Ipsen A., Seebens A., Gottsche M., Panning M., Drexler J.F., Petersen N., Annan A., Grywna K., Muller M. (2008). Detection and prevalence patterns of group I coronaviruses in bats, northern Germany. Emerg. Infect. Dis..

[49] Fischer K., Zeus V., Kwasnitschka L., Kerth G., Haase M., Groschup M.H., Balkema-Buschmann A. (2016). Insectivorous bats carry host specific astroviruses and coronaviruses across different regions in Germany. Infect. Genet. Evol..

[50] Falcon A., Vazquez-Moron S., Casas I., Aznar C., Ruiz G., Pozo F., Perez-Brena P., Juste J., Ibanez C., Garin I. (2011). Detection of alpha and betacoronaviruses in multiple Iberian bat species. Arch. Virol..

[51] Ar Gouilh M., Puechmaille S.J., Diancourt L., Vandenbogaert M., Serra-Cobo J., Lopez Roig M., Brown P., Moutou F., Caro V., Vabret A. (2018). SARS-CoV related Betacoronavirus and diverse Alphacoronavirus members found in western old-world. Virology.

[52] Goffard A., Demanche C., Arthur L., Pincon C., Michaux J., Dubuisson J. (2015). Alphacoronaviruses Detected in French Bats Are Phylogeographically Linked to Coronaviruses of European Bats. Viruses.

[53] Kivisto I., Tidenberg E.M., Lilley T., Suominen K., Forbes K.M., Vapalahti O., Huovilainen A., Sironen T. (2020). First Report of Coronaviruses in Northern European Bats. Vector Borne Zoonotic Dis..

[54] Rizzo F., Edenborough K.M., Toffoli R., Culasso P., Zoppi S., Dondo A., Robetto S., Rosati S., Lander A., Kurth A. (2017). Coronavirus and paramyxovirus in bats from Northwest Italy. BMC Vet. Res..

[55] De Sabato L., Lelli D., Faccin F., Canziani S., Di Bartolo I., Vaccari G., Moreno A. (2019). Full genome characterization of two novel Alpha-coronavirus species from Italian bats. Virus Res..

[56] De Benedictis P., Marciano S., Scaravelli D., Priori P., Zecchin B., Capua I., Monne I., Cattoli G. (2014). Alpha and lineage C betaCoV infections in Italian bats. Virus Genes.

[57] Lelli D., Papetti A., Sabelli C., Rosti E., Moreno A., Boniotti M.B. (2013). Detection of coronaviruses in bats of various species in Italy. Viruses.

[58] August T.A., Mathews F., Nunn M.A. (2012). Alphacoronavirus detected in bats in the United Kingdom. Vector Borne Zoonotic Dis..

[59] Lazov C.M., Chriel M., Baagoe H.J., Fjederholt E., Deng Y., Kooi E.A., Belsham G.J., Botner A., Rasmussen T.B. (2018). Detection and Characterization of Distinct Alphacoronaviruses in Five Different Bat Species in Denmark. Viruses.

[60] Pauly M., Pir J.B., Loesch C., Sausy A., Snoeck C.J., Hubschen J.M., Muller C.P. (2017). Novel Alphacoronaviruses and Paramyxoviruses Cocirculate with Type 1 and Severe Acute Respiratory System (SARS)-Related Betacoronaviruses in Synanthropic Bats of Luxembourg. Appl. Environ. Microbiol..

[61] Drexler J.F., Gloza-Rausch F., Glende J., Corman V.M., Muth D., Goettsche M., Seebens A., Niedrig M., Pfefferle S., Yordanov S. (2010). Genomic characterization of severe acute respiratory syndrome-related coronavirus in European bats and classification of coronaviruses based on partial RNA-dependent RNA polymerase gene sequences. J. Virol..

[62] Rydell J., Bach L., Dubourg-Savage M.J., Green M., Rodrigues L., Hedenström A. (2010). Mortality of bats at wind turbines links to nocturnal insect migration?. Eur. J. Wildl. Res..

[63] Annan A., Baldwin H.J., Corman V.M., Klose S.M., Owusu M., Nkrumah E.E., Badu E.K., Anti P., Agbenyega O., Meyer B. (2013). Human betacoronavirus 2c EMC/2012-related viruses in bats, Ghana and Europe. Emerg. Infect. Dis..

[64] Rihtaric D., Hostnik P., Steyer A., Grom J., Toplak I. (2010). Identification of SARS-like coronaviruses in horseshoe bats (*Rhinolophus hipposideros*) in Slovenia. Arch. Virol..

[65] Reusken C.B., Lina P.H., Pielaat A., de Vries A., Dam-Deisz C., Adema J., Drexler J.F., Drosten C., Kooi E.A. (2010). Circulation of group 2 coronaviruses in a bat species common to urban areas in Western Europe. Vector Borne Zoonotic Dis..

[66] Moreno A., Lelli D., De Sabato L., Zaccaria G., Boni A., Sozzi E., Prosperi A., Lavazza A., Cella E., Castrucci M.R. (2017). Detection and full genome characterization of two beta CoV viruses related to Middle East respiratory syndrome from bats in Italy. Virol. J..

[67] Balboni A., Palladini A., Bogliani G., Battilani M. (2011). Detection of a virus related to betacoronaviruses in Italian greater horseshoe bats. Epidemiol. Infect..

[68] Lecis R., Mucedda M., Pidinchedda E., Pittau M., Alberti A. (2019). Molecular identification of Betacoronavirus in bats from Sardinia (Italy): First detection and phylogeny. Virus Genes.

[69] Negredo A., Palacios G., Vazquez-Moron S., Gonzalez F., Dopazo H., Molero F., Juste J., Quetglas J., Savji N., de la Cruz Martinez M. (2011). Discovery of an ebolavirus-like filovirus in Europe. PLoS Pathog..

[70] Cadar D., Becker N., de Mendoza Campos R., Borstler J., Jost H., Schmidt-Chanasit J. (2014). Usutu virus in bats, Germany, 2013. Emerg. Infect. Dis..

[71] Strakova P., Dufkova L., Sirmarova J., Salat J., Bartonicka T., Klempa B., Pfaff F., Hoper D., Hoffmann B., Ulrich R.G. (2017). Novel hantavirus identified in European bat species *Nyctalus noctula*. Infect. Genet. Evol..

[72] Drexler J.F., Seelen A., Corman V.M., Fumie Tateno A., Cottontail V., Melim Zerbinati R., Gloza-Rausch F., Klose S.M., Adu-Sarkodie Y., Oppong S.K. (2012). Bats worldwide carry hepatitis E virus-related viruses that form a putative novel genus within the family Hepeviridae. J. Virol..

[73] Wibbelt G., Kurth A., Yasmum N., Bannert M., Nagel S., Nitsche A., Ehlers B. (2007). Discovery of herpesviruses in bats. J. Gen. Virol..

[74] Pozo F., Juste J., Vazquez-Moron S., Aznar-Lopez C., Ibanez C., Garin I., Aihartza J., Casas I., Tenorio A., Echevarria J.E. (2016). Identification of Novel Betaherpesviruses in Iberian Bats Reveals Parallel Evolution. PLoS ONE.

[75] Molnar V., Janoska M., Harrach B., Glavits R., Palmai N., Rigo D., Sos E., Liptovszky M. (2008). Detection of a novel bat gammaherpesvirus in Hungary. Acta Vet. Hung..

[76] Garcia-Perez R., Ibanez C., Godinez J.M., Arechiga N., Garin I., Perez-Suarez G., de Paz O., Juste J., Echevarria J.E., Bravo I.G. (2014). Novel papillomaviruses in free-ranging Iberian bats: No virus-host co-evolution, no strict host specificity, and hints for recombination. Genome Biol. Evol..

[77] Kurth A., Kohl C., Brinkmann A., Ebinger A., Harper J.A., Wang L.F., Muhldorfer K., Wibbelt G. (2012). Novel paramyxoviruses in free-ranging European bats. PLoS ONE.

[78] Drexler J.F., Corman V.M., Muller M.A., Maganga G.D., Vallo P., Binger T., Gloza-Rausch F., Cottontail V.M., Rasche A., Yordanov S. (2012). Bats host major mammalian paramyxoviruses. Nat. Commun..

[79] Drexler J.F., Corman V.M., Lukashev A.N., van den Brand J.M., Gmyl A.P., Brunink S., Rasche A., Seggewibeta N., Feng H., Leijten L.M. (2015). Evolutionary origins of hepatitis A virus in small mammals. Proc. Natl. Acad. Sci. USA.

[80] Vidovszky M.Z., Tan Z., Carr M.J., Boldogh S., Harrach B., Gonzalez G. (2020). Bat-borne polyomaviruses in Europe reveal an evolutionary history of intrahost divergence with horseshoe bats distributed across the African and Eurasian continents. J. Gen. Virol..

[81] Lelli D., Lavazza A., Prosperi A., Sozzi E., Faccin F., Baioni L., Trogu T., Cavallari G.L., Mauri M., Gibellini A.M. (2019). Hypsugopoxvirus: A Novel Poxvirus Isolated from Hypsugo savii in Italy. Viruses.

[82] Kohl C., Lesnik R., Brinkmann A., Ebinger A., Radonic A., Nitsche A., Muhldorfer K., Wibbelt G., Kurth A. (2012). Isolation and characterization of three mammalian orthoreoviruses from European bats. PLoS ONE.

[83] Lelli D., Moreno A., Lavazza A., Bresaola M., Canelli E., Boniotti M.B., Cordioli P. (2013). Identification of Mammalian orthoreovirus type 3 in Italian bats. Zoonoses Public Health.

[84] Naglic T., Rihtaric D., Hostnik P., Toplak N., Koren S., Kuhar U., Jamnikar-Ciglenecki U., Kutnjak D., Steyer A. (2018). Identification of novel reassortant mammalian orthoreoviruses from bats in Slovenia. BMC Vet. Res..

[85] Simsek C., Corman V.M., Everling H.U., Lukashev A.N., Rasche A., Maganga G.D., Binger T., Jansen D., Beller L., Deboutte W. (2021). At Least Seven Distinct Rotavirus Genotype Constellations in Bats with Evidence of Reassortment and Zoonotic Transmissions. mBio.

[86] Nokireki T., Tammiranta N., Kokkonen U.M., Kantala T., Gadd T. (2018). Tentative novel lyssavirus in a bat in Finland. Transbound. Emerg. Dis..

[87] Picard-Meyer E., Beven V., Hirchaud E., Guillaume C., Larcher G., Robardet E., Servat A., Blanchard Y., Cliquet F. (2019). Lleida Bat Lyssavirus isolation in *Miniopterus schreibersii* in France. Zoonoses Public Health.

[88] Mingo-Casas P., Sandonis V., Obon E., Berciano J.M., Vazquez-Moron S., Juste J., Echevarria J.E. (2018). First cases of European bat lyssavirus type 1 in Iberian serotine bats: Implications for the molecular epidemiology of bat rabies in Europe. PLoS Neglect. Trop. Dis..

[89] Nokireki T., Sironen T., Smura T., Karkamo V., Sihvonen L., Gadd T. (2017). Second case of European bat lyssavirus type 2 detected in a Daubenton’s bat in Finland. Acta Vet. Scand..

[90] Lelli D., Prosperi A., Moreno A., Chiapponi C., Gibellini A.M., De Benedictis P., Leopardi S., Sozzi E., Lavazza A. (2018). Isolation of a novel Rhabdovirus from an insectivorous bat (*Pipistrellus kuhlii*) in Italy. Virol. J..

[91] Moldal T., Vikoren T., Cliquet F., Marston D.A., van der Kooij J., Madslien K., Orpetveit I. (2017). First detection of European bat lyssavirus type 2 (EBLV-2) in Norway. BMC Vet. Res..

[92] Bourhy H., Kissi B., Lafon M., Sacramento D., Tordo N. (1992). Antigenic and molecular characterization of bat rabies virus in Europe. J. Clin. Microbiol..

[93] Fooks A.R., Brookes S.M., Johnson N., McElhinney L.M., Hutson A.M. (2003). European bat lyssaviruses: An emerging zoonosis. Epidemiol. Infect..

[94] Muller T., Johnson N., Freuling C.M., Fooks A.R., Selhorst T., Vos A. (2007). Epidemiology of bat rabies in Germany. Arch. Virol..

[95] Picard-Meyer E., Barrat J., Tissot E., Barrat M.J., Bruyere V., Cliquet F. (2004). Genetic analysis of European bat lyssavirus type 1 isolates from France. Vet. Rec..

[96] Freuling C.M., Beer M., Conraths F.J., Finke S., Hoffmann B., Keller B., Kliemt J., Mettenleiter T.C., Muhlbach E., Teifke J.P. (2011). Novel lyssavirus in Natterer’s bat, Germany. Emerg. Infect. Dis..

[97] Jakava-Viljanen M., Lilley T., Kyheroinen E.M., Huovilainen A. (2010). First encounter of European bat lyssavirus type 2 (EBLV-2) in a bat in Finland. Epidemiol. Infect..

[98] Vazquez-Moron S., Juste J., Ibanez C., Berciano J.M., Echevarria J.E. (2011). Phylogeny of European bat Lyssavirus 1 in Eptesicus isabellinus bats, Spain. Emerg. Infect. Dis..

[99] Smreczak M., Orlowska A., Marzec A., Trebas P., Muller T., Freuling C.M., Zmudzinski J.F. (2018). Bokeloh bat lyssavirus isolation in a Natterer’s bat, Poland. Zoonoses Public Health.

[100] Picard-Meyer E., Servat A., Robardet E., Moinet M., Borel C., Cliquet F. (2013). Isolation of Bokeloh bat lyssavirus in Myotis nattereri in France. Arch. Virol..

[101] Schatz J., Fooks A.R., McElhinney L., Horton D., Echevarria J., Vazquez-Moron S., Kooi E.A., Rasmussen T.B., Muller T., Freuling C.M. (2013). Bat rabies surveillance in Europe. Zoonoses Public Health.

[102] Freuling C.M., Abendroth B., Beer M., Fischer M., Hanke D., Hoffmann B., Hoper D., Just F., Mettenleiter T.C., Schatz J. (2013). Molecular diagnostics for the detection of Bokeloh bat lyssavirus in a bat from Bavaria, Germany. Virus Res..

[103] Aznar-Lopez C., Vazquez-Moron S., Marston D.A., Juste J., Ibanez C., Berciano J.M., Salsamendi E., Aihartza J., Banyard A.C., McElhinney L. (2013). Detection of rhabdovirus viral RNA in oropharyngeal swabs and ectoparasites of Spanish bats. J. Gen. Virol..

[104] Calvelage S., Tammiranta N., Nokireki T., Gadd T., Eggerbauer E., Zaeck L.M., Potratz M., Wylezich C., Hoper D., Muller T. (2021). Genetic and Antigenetic Characterization of the Novel Kotalahti Bat Lyssavirus (KBLV). Viruses.

[105] Arechiga Ceballos N., Vazquez Moron S., Berciano J.M., Nicolas O., Aznar Lopez C., Juste J., Rodriguez Nevado C., Aguilar Setien A., Echevarria J.E. (2013). Novel lyssavirus in bat, Spain. Emerg. Infect. Dis..

[106] Banyard A.C., Selden D., Wu G., Thorne L., Jennings D., Marston D., Finke S., Freuling C.M., Muller T., Echevarria J.E. (2018). Isolation, antigenicity and immunogenicity of Lleida bat lyssavirus. J. Gen. Virol..

[107] Kuzmin I.V., Hughes G.J., Botvinkin A.D., Orciari L.A., Rupprecht C.E. (2005). Phylogenetic relationships of Irkut and West Caucasian bat viruses within the Lyssavirus genus and suggested quantitative criteria based on the N gene sequence for lyssavirus genotype definition. Virus Res..

[108] Van der Poel W.H., Van der Heide R., Verstraten E.R., Takumi K., Lina P.H., Kramps J.A. (2005). European bat lyssaviruses, The Netherlands. Emerg. Infect. Dis..

[109] Delmas O., Holmes E.C., Talbi C., Larrous F., Dacheux L., Bouchier C., Bourhy H. (2008). Genomic diversity and evolution of the lyssaviruses. PLoS ONE.

[110] Dacheux L., Berthet N., Dissard G., Holmes E.C., Delmas O., Larrous F., Guigon G., Dickinson P., Faye O., Sall A.A. (2010). Application of broad-spectrum resequencing microarray for genotyping rhabdoviruses. J. Virol..

[111] Badrane H., Bahloul C., Perrin P., Tordo N. (2001). Evidence of two Lyssavirus phylogroups with distinct pathogenicity and immunogenicity. J. Virol..

[112] ICTV (2021). International Committee on Taxonomy of Viruses (ICTV). https://talk.ictvonline.org/taxonomy/.

[113] Wang L.F., Shi Z., Zhang S., Field H., Daszak P., Eaton B.T. (2006). Review of bats and SARS. Emerg. Infect. Dis..

[114] Banerjee A., Kulcsar K., Misra V., Frieman M., Mossman K. (2019). Bats and Coronaviruses. Viruses.

[115] Cui J., Li F., Shi Z.L. (2019). Origin and evolution of pathogenic coronaviruses. Nat. Rev. Microbiol..

[116] Drosten C., Gunther S., Preiser W., van der Werf S., Brodt H.R., Becker S., Rabenau H., Panning M., Kolesnikova L., Fouchier R.A. (2003). Identification of a novel coronavirus in patients with severe acute respiratory syndrome. N. Engl. J. Med..

[117] Fouchier R.A., Kuiken T., Schutten M., van Amerongen G., van Doornum G.J., van den Hoogen B.G., Peiris M., Lim W., Stohr K., Osterhaus A.D. (2003). Aetiology: Koch’s postulates fulfilled for SARS virus. Nature.

[118] Poon L.L., Guan Y., Nicholls J.M., Yuen K.Y., Peiris J.S. (2004). The aetiology, origins, and diagnosis of severe acute respiratory syndrome. Lancet Infect. Dis..

[119] Ge X.Y., Li J.L., Yang X.L., Chmura A.A., Zhu G., Epstein J.H., Mazet J.K., Hu B., Zhang W., Peng C. (2013). Isolation and characterization of a bat SARS-like coronavirus that uses the ACE2 receptor. Nature.

[120] Menachery V.D., Yount B.L., Debbink K., Agnihothram S., Gralinski L.E., Plante J.A., Graham R.L., Scobey T., Ge X.Y., Donaldson E.F. (2015). A SARS-like cluster of circulating bat coronaviruses shows potential for human emergence. Nat. Med..

[121] Lau S.K., Woo P.C., Li K.S., Huang Y., Tsoi H.W., Wong B.H., Wong S.S., Leung S.Y., Chan K.H., Yuen K.Y. (2005). Severe acute respiratory syndrome coronavirus-like virus in Chinese horseshoe bats. Proc. Natl. Acad. Sci. USA.

[122] Hu B., Zeng L.P., Yang X.L., Ge X.Y., Zhang W., Li B., Xie J.Z., Shen X.R., Zhang Y.Z., Wang N. (2017). Discovery of a rich gene pool of bat SARS-related coronaviruses provides new insights into the origin of SARS coronavirus. PLoS Pathog..

[123] Al-Salihi K.A., Khalaf J.M. (2021). The emerging SARS-CoV, MERS-CoV, and SARS-CoV-2: An insight into the viruses zoonotic aspects. Vet. World.

[124] Zaki A.M., van Boheemen S., Bestebroer T.M., Osterhaus A.D., Fouchier R.A. (2012). Isolation of a novel coronavirus from a man with pneumonia in Saudi Arabia. N. Engl. J. Med..

[125] Mohd H.A., Al-Tawfiq J.A., Memish Z.A. (2016). Middle East Respiratory Syndrome Coronavirus (MERS-CoV) origin and animal reservoir. Virol. J..

[126] De Wit E., van Doremalen N., Falzarano D., Munster V.J. (2016). SARS and MERS: Recent insights into emerging coronaviruses. Nat. Rev. Microbiol..

[127] Wang Q., Qi J., Yuan Y., Xuan Y., Han P., Wan Y., Ji W., Li Y., Wu Y., Wang J. (2014). Bat origins of MERS-CoV supported by bat coronavirus HKU4 usage of human receptor CD26. Cell Host Microbe.

[128] Corman V.M., Muth D., Niemeyer D., Drosten C. (2018). Hosts and Sources of Endemic Human Coronaviruses. Adv. Virus Res..

[129] Andersen K.G., Rambaut A., Lipkin W.I., Holmes E.C., Garry R.F. (2020). The proximal origin of SARS-CoV-2. Nat. Med..

[130] Wu F., Zhao S., Yu B., Chen Y.M., Wang W., Song Z.G., Hu Y., Tao Z.W., Tian J.H., Pei Y.Y. (2020). A new coronavirus associated with human respiratory disease in China. Nature.

[131] Ge X.Y., Wang N., Zhang W., Hu B., Li B., Zhang Y.Z., Zhou J.H., Luo C.M., Yang X.L., Wu L.J. (2016). Coexistence of multiple coronaviruses in several bat colonies in an abandoned mineshaft. Virol. Sin..

[132] Li W., Wong S.K., Li F., Kuhn J.H., Huang I.C., Choe H., Farzan M. (2006). Animal origins of the severe acute respiratory syndrome coronavirus: Insight from ACE2-S-protein interactions. J. Virol..

[133] Lam T.T., Jia N., Zhang Y.W., Shum M.H., Jiang J.F., Zhu H.C., Tong Y.G., Shi Y.X., Ni X.B., Liao Y.S. (2020). Identifying SARS-CoV-2-related coronaviruses in Malayan pangolins. Nature.

[134] Zhang T., Wu Q., Zhang Z. (2020). Pangolin homology associated with 2019-nCoV. bioRxiv.

[135] Siegert R., Shu H.L., Slenczka W. (1968). Isolation and identification of the “Marbury virus”. Ger. Med. Mon..

[136] Slenczka W., Klenk H.D. (2007). Forty years of Marburg virus. J. Infect. Dis..

[137] Peterson A.T., Carroll D.S., Mills J.N., Johnson K.M. (2004). Potential mammalian filovirus reservoirs. Emerg. Infect. Dis..

[138] Pourrut X., Souris M., Towner J.S., Rollin P.E., Nichol S.T., Gonzalez J.P., Leroy E. (2009). Large serological survey showing cocirculation of Ebola and Marburg viruses in Gabonese bat populations, and a high seroprevalence of both viruses in *Rousettus aegyptiacus*. BMC Infect. Dis..

[139] Adjemian J., Farnon E.C., Tschioko F., Wamala J.F., Byaruhanga E., Bwire G.S., Kansiime E., Kagirita A., Ahimbisibwe S., Katunguka F. (2011). Outbreak of Marburg hemorrhagic fever among miners in Kamwenge and Ibanda Districts, Uganda, 2007. J. Infect. Dis..

[140] Amman B.R., Carroll S.A., Reed Z.D., Sealy T.K., Balinandi S., Swanepoel R., Kemp A., Erickson B.R., Comer J.A., Campbell S. (2012). Seasonal pulses of Marburg virus circulation in juvenile *Rousettus aegyptiacus* bats coincide with periods of increased risk of human infection. PLoS Pathog..

[141] Brauburger K., Hume A.J., Muhlberger E., Olejnik J. (2012). Forty-five years of Marburg virus research. Viruses.

[142] Timen A., Koopmans M.P., Vossen A.C., van Doornum G.J., Gunther S., van den Berkmortel F., Verduin K.M., Dittrich S., Emmerich P., Osterhaus A.D. (2009). Response to imported case of Marburg hemorrhagic fever, The Netherlands. Emerg. Infect. Dis..

[143] Formenty P., Libama F., Epelboin A., Allarangar Y., Leroy E., Moudzeo H., Tarangonia P., Molamou A., Lenzi M., Ait-Ikhlef K. (2003). Outbreak of Ebola hemorrhagic fever in the Republic of the Congo, 2003: A new strategy?. Med. Trop..

[144] Onyango C.O., Opoka M.L., Ksiazek T.G., Formenty P., Ahmed A., Tukei P.M., Sang R.C., Ofula V.O., Konongoi S.L., Coldren R.L. (2007). Laboratory diagnosis of Ebola hemorrhagic fever during an outbreak in Yambio, Sudan, 2004. J. Infect. Dis..

[145] Emond R.T., Evans B., Bowen E.T., Lloyd G. (1977). A case of Ebola virus infection. Br. Med. J..

[146] Carroll M.W., Matthews D.A., Hiscox J.A., Elmore M.J., Pollakis G., Rambaut A., Hewson R., Garcia-Dorival I., Bore J.A., Koundouno R. (2015). Temporal and spatial analysis of the 2014–2015 Ebola virus outbreak in West Africa. Nature.

[147] Mari Saez A., Weiss S., Nowak K., Lapeyre V., Zimmermann F., Dux A., Kuhl H.S., Kaba M., Regnaut S., Merkel K. (2015). Investigating the zoonotic origin of the West African Ebola epidemic. EMBO Mol. Med..

[148] Karan L.S., Makenov M.T., Korneev M.G., Sacko N., Boumbaly S., Yakovlev S.A., Kourouma K., Bayandin R.B., Gladysheva A.V., Shipovalov A.V. (2019). Bombali Virus in Mops condylurus Bats, Guinea. Emerg. Infect. Dis..

[149] Forbes K.M., Webala P.W., Jaaskelainen A.J., Abdurahman S., Ogola J., Masika M.M., Kivisto I., Alburkat H., Plyusnin I., Levanov L. (2019). Bombali Virus in *Mops condylurus* Bat, Kenya. Emerg. Infect. Dis..

[150] Goldstein T., Anthony S.J., Gbakima A., Bird B.H., Bangura J., Tremeau-Bravard A., Belaganahalli M.N., Wells H.L., Dhanota J.K., Liang E. (2018). The discovery of Bombali virus adds further support for bats as hosts of ebolaviruses. Nat. Microbiol..

[151] Jayme S.I., Field H.E., de Jong C., Olival K.J., Marsh G., Tagtag A.M., Hughes T., Bucad A.C., Barr J., Azul R.R. (2015). Molecular evidence of Ebola Reston virus infection in Philippine bats. Virol. J..

[152] Yang X.L., Zhang Y.Z., Jiang R.D., Guo H., Zhang W., Li B., Wang N., Wang L., Waruhiu C., Zhou J.H. (2017). Genetically Diverse Filoviruses in *Rousettus* and *Eonycteris* spp. Bats, China, 2009 and 2015. Emerg. Infect. Dis..

[153] Ramirez de Arellano E., Sanchez-Lockhart M., Perteguer M.J., Bartlett M., Ortiz M., Campioli P., Hernandez A., Gonzalez J., Garcia K., Ramos M. (2019). First Evidence of Antibodies Against Lloviu Virus in Schreiber’s Bent-Winged Insectivorous Bats Demonstrate a Wide Circulation of the Virus in Spain. Viruses.

[154] Appleton B.R., McKenzie J.A., Christidis L. (2004). Molecular systematics and biogeography of the bent-wing bat complex *Miniopterus schreibersii* (Kuhl, 1817) (Chiroptera: Vespertilionidae). Mol. Phylogenet. Evol..

[155] Ain-Najwa M.Y., Yasmin A.R., Arshad S.S., Omar A.R., Abu J., Kumar K., Mohammed H.O., Natasha J.A., Mohammed M.N., Bande F. (2020). Exposure to Zoonotic West Nile Virus in Long-Tailed Macaques and Bats in Peninsular Malaysia. Animals.

[156] Paul S.D., Rajagopalan P.K., Sreenivasan M.A. (1970). Isolation of the West Nile virus from the frugivorous bat, *Rousettus leschenaulti*. Indian J. Med. Res..

[157] Torres-Castro M., Noh-Pech H., Hernandez-Betancourt S., Pelaez-Sanchez R., Lugo-Caballero C., Puerto F.I. (2021). West Nile and Zika viruses in bats from a suburban area of Merida, Yucatan, Mexico. Zoonoses Public Health.

[158] Bunde J.M., Heske E.J., Mateus-Pinilla N.E., Hofmann J.E., Novak R.J. (2006). A survey for West Nile virus in bats from Illinois. J. Wildl. Dis..

[159] Davis A., Bunning M., Gordy P., Panella N., Blitvich B., Bowen R. (2005). Experimental and natural infection of North American bats with West Nile virus. Am. J. Trop. Med. Hyg..

[160] Pilipski J.D., Pilipskl L.M., Risley L.S. (2004). West Nile virus antibodies in bats from New Jersey and New York. J. Wildl. Dis..

[161] Allen R., Taylor S.K., Sulkin S.E. (1970). Studies of arthropod-borne virus infections in Chiroptera. 8. Evidence of natural St. Louis encephalitis virus infection in bats. Am. J. Trop. Med. Hyg..

[162] Cui J., Counor D., Shen D., Sun G., He H., Deubel V., Zhang S. (2008). Detection of Japanese encephalitis virus antibodies in bats in Southern China. Am. J. Trop. Med. Hyg..

[163] Epstein J.H., Quan P.L., Briese T., Street C., Jabado O., Conlan S., Ali Khan S., Verdugo D., Hossain M.J., Hutchison S.K. (2010). Identification of GBV-D, a novel GB-like flavivirus from old world frugivorous bats (*Pteropus giganteus*) in Bangladesh. PLoS Pathog..

[164] Kuno G., Chang G.J. (2006). Characterization of Sepik and Entebbe bat viruses closely related to yellow fever virus. Am. J. Trop. Med. Hyg..

[165] Tajima S., Takasaki T., Matsuno S., Nakayama M., Kurane I. (2005). Genetic characterization of Yokose virus, a flavivirus isolated from the bat in Japan. Virology.

[166] Watanabe S., Omatsu T., Miranda M.E., Masangkay J.S., Ueda N., Endo M., Kato K., Tohya Y., Yoshikawa Y., Akashi H. (2010). Epizootology and experimental infection of Yokose virus in bats. Comp. Immunol. Microbiol. Infect. Dis..

[167] Weaver S.C., Barrett A.D. (2004). Transmission cycles, host range, evolution and emergence of arboviral disease. Nat. Rev. Microbiol..

[168] Weissenbock H., Bakonyi T., Rossi G., Mani P., Nowotny N. (2013). Usutu virus, Italy, 1996. Emerg. Infect. Dis..

[169] Kruger D.H., Schonrich G., Klempa B. (2011). Human pathogenic hantaviruses and prevention of infection. Hum. Vaccines.

[170] Arai S., Nguyen S.T., Boldgiv B., Fukui D., Araki K., Dang C.N., Ohdachi S.D., Nguyen N.X., Pham T.D., Boldbaatar B. (2013). Novel bat-borne hantavirus, Vietnam. Emerg. Infect. Dis..

[171] De Araujo J., Thomazelli L.M., Henriques D.A., Lautenschalager D., Ometto T., Dutra L.M., Aires C.C., Favorito S., Durigon E.L. (2012). Detection of hantavirus in bats from remaining rain forest in Sao Paulo, Brazil. BMC Res. Notes.

[172] Guo W.P., Lin X.D., Wang W., Tian J.H., Cong M.L., Zhang H.L., Wang M.R., Zhou R.H., Wang J.B., Li M.H. (2013). Phylogeny and origins of hantaviruses harbored by bats, insectivores, and rodents. PLoS Pathog..

[173] Sumibcay L., Kadjo B., Gu S.H., Kang H.J., Lim B.K., Cook J.A., Song J.W., Yanagihara R. (2012). Divergent lineage of a novel hantavirus in the banana pipistrelle (*Neoromicia nanus*) in Cote d’Ivoire. Virol. J..

[174] Weiss S., Witkowski P.T., Auste B., Nowak K., Weber N., Fahr J., Mombouli J.V., Wolfe N.D., Drexler J.F., Drosten C. (2012). Hantavirus in bat, Sierra Leone. Emerg. Infect. Dis..

[175] Arai S., Kikuchi F., Bawm S., Son N.T., Lin K.S., Tu V.T., Aoki K., Tsuchiya K., Tanaka-Taya K., Morikawa S. (2019). Molecular Phylogeny of Mobatviruses (Hantaviridae) in Myanmar and Vietnam. Viruses.

[176] Charrel R.N., Gallian P., Navarro-Mari J.M., Nicoletti L., Papa A., Sanchez-Seco M.P., Tenorio A., de Lamballerie X. (2005). Emergence of Toscana virus in Europe. Emerg. Infect. Dis..

[177] Grobbelaar A.A., Weyer J., Leman P.A., Kemp A., Paweska J.T., Swanepoel R. (2011). Molecular epidemiology of Rift Valley fever virus. Emerg. Infect. Dis..

[178] Boiro I., Konstaninov O.K., Numerov A.D. (1987). Isolation of Rift Valley fever virus from bats in the Republic of Guinea. Bull. Soc. Pathol. Exot. Filiales.

[179] Yu X.J., Liang M.F., Zhang S.Y., Liu Y., Li J.D., Sun Y.L., Zhang L., Zhang Q.F., Popov V.L., Li C. (2011). Fever with thrombocytopenia associated with a novel bunyavirus in China. N. Engl. J. Med..

[180] Zhang Y.Z., He Y.W., Dai Y.A., Xiong Y., Zheng H., Zhou D.J., Li J., Sun Q., Luo X.L., Cheng Y.L. (2012). Hemorrhagic fever caused by a novel Bunyavirus in China: Pathogenesis and correlates of fatal outcome. Clin. Infect. Dis..

[181] Park S.W., Han M.G., Yun S.M., Park C., Lee W.J., Ryou J. (2014). Severe fever with thrombocytopenia syndrome virus, South Korea, 2013. Emerg. Infect. Dis..

[182] Takahashi T., Maeda K., Suzuki T., Ishido A., Shigeoka T., Tominaga T., Kamei T., Honda M., Ninomiya D., Sakai T. (2014). The first identification and retrospective study of Severe Fever with Thrombocytopenia Syndrome in Japan. J. Infect. Dis..

[183] McMullan L.K., Folk S.M., Kelly A.J., MacNeil A., Goldsmith C.S., Metcalfe M.G., Batten B.C., Albarino C.G., Zaki S.R., Rollin P.E. (2012). A new phlebovirus associated with severe febrile illness in Missouri. N. Engl. J. Med..

[184] Savage H.M., Godsey M.S., Lambert A., Panella N.A., Burkhalter K.L., Harmon J.R., Lash R.R., Ashley D.C., Nicholson W.L. (2013). First detection of heartland virus (Bunyaviridae: Phlebovirus) from field collected arthropods. Am. J. Trop. Med. Hyg..

[185] Mourya D.T., Yadav P.D., Basu A., Shete A., Patil D.Y., Zawar D., Majumdar T.D., Kokate P., Sarkale P., Raut C.G. (2014). Malsoor virus, a novel bat phlebovirus, is closely related to severe fever with thrombocytopenia syndrome virus and heartland virus. J. Virol..

[186] Walker P.J., Siddell S.G., Lefkowitz E.J., Mushegian A.R., Adriaenssens E.M., Dempsey D.M., Dutilh B.E., Harrach B., Harrison R.L., Hendrickson R.C. (2020). Changes to virus taxonomy and the Statutes ratified by the International Committee on Taxonomy of Viruses (2020). Arch. Virol..

[187] MacLachlan N.J., Dubovi E.J. (2016). Fenner’s Veterinary Virology.

[188] Muller M.A., Devignot S., Lattwein E., Corman V.M., Maganga G.D., Gloza-Rausch F., Binger T., Vallo P., Emmerich P., Cottontail V.M. (2016). Evidence for widespread infection of African bats with Crimean-Congo hemorrhagic fever-like viruses. Sci. Rep..

[189] Walker P.J., Widen S.G., Wood T.G., Guzman H., Tesh R.B., Vasilakis N. (2016). A Global Genomic Characterization of Nairoviruses Identifies Nine Discrete Genogroups with Distinctive Structural Characteristics and Host-Vector Associations. Am. J. Trop. Med. Hyg..

[190] Kuhn J.H., Wiley M.R., Rodriguez S.E., Bao Y., Prieto K., Travassos da Rosa A.P., Guzman H., Savji N., Ladner J.T., Tesh R.B. (2016). Genomic Characterization of the Genus *Nairovirus* (Family Bunyaviridae). Viruses.

[191] Quillien M.C., Monnat J.Y., Le Lay G., Le Goff F., Hardy E., Chastel C. (1986). Avalon virus, Sakhalin group (*Nairovirus*, Bunyaviridae) from the seabird tick Ixodes (Ceratixodes) uriae White 1852 in France. Acta Virol..

[192] Lvov D.K., Karas F.R., Timofeev E.M., Tsyrkin Y.M., Vargina S.G., Veselovskaya O.V., Osipova N.Z., Grebenyuk Y.I., Gromashevski V.L., Steblyanko S.N. (1973). “Issyk-Kul” virus, a new arbovirus isolated from bats and *Argas* (Carios) *vespertilionis* (Latr., 1802) in the Kirghiz, S.S.R. Brief report. Arch. Gesamte Virusforsch..

[193] L’Vov D.K., Kostiukov M.A., Daniiarov O.A., Tukhtaev T.M., Sherikov B.K. (1984). Outbreak of arbovirus infection in the Tadzhik SSR due to the Issyk-Kul virus (Issyk-Kul fever). Vopr. Virusol..

[194] Kapoor A., Tesh R.B., Duraisamy R., Popov V.L., Travassos da Rosa A.P.A., Lipkin W.I. (2013). A novel mosquito-borne *Orbivirus* species found in South-east Asia. J. Gen. Virol..

[195] Weiss S., Dabrowski P.W., Kurth A., Leendertz S.A.J., Leendertz F.H. (2017). A novel Coltivirus-related virus isolated from free-tailed bats from Cote d’Ivoire is able to infect human cells in vitro. Virol. J..

[196] Gard G.P., Marshall I.D. (1973). Nelson Bay virus. A novel reovirus. Arch. Gesamte Virusforsch..

[197] Pritchard L.I., Chua K.B., Cummins D., Hyatt A., Crameri G., Eaton B.T., Wang L.F. (2006). Pulau virus; A new member of the Nelson Bay orthoreovirus species isolated from fruit bats in Malaysia. Arch. Virol..

[198] Chua K.B., Crameri G., Hyatt A., Yu M., Tompang M.R., Rosli J., McEachern J., Crameri S., Kumarasamy V., Eaton B.T. (2007). A previously unknown reovirus of bat origin is associated with an acute respiratory disease in humans. Proc. Natl. Acad. Sci. USA.

[199] Du L., Lu Z., Fan Y., Meng K., Jiang Y., Zhu Y., Wang S., Gu W., Zou X., Tu C. (2010). Xi River virus, a new bat reovirus isolated in southern China. Arch. Virol..

[200] Thalmann C.M., Cummins D.M., Yu M., Lunt R., Pritchard L.I., Hansson E., Crameri S., Hyatt A., Wang L.F. (2010). Broome virus, a new fusogenic Orthoreovirus species isolated from an Australian fruit bat. Virology.

[201] Cheng P., Lau C.S., Lai A., Ho E., Leung P., Chan F., Wong A., Lim W. (2009). A novel reovirus isolated from a patient with acute respiratory disease. J. Clin. Virol..

[202] Chua K.B., Voon K., Crameri G., Tan H.S., Rosli J., McEachern J.A., Suluraju S., Yu M., Wang L.F. (2008). Identification and characterization of a new orthoreovirus from patients with acute respiratory infections. PLoS ONE.

[203] Chua K.B., Voon K., Yu M., Keniscope C., Abdul Rasid K., Wang L.F. (2011). Investigation of a potential zoonotic transmission of orthoreovirus associated with acute influenza-like illness in an adult patient. PLoS ONE.

[204] Decaro N., Campolo M., Desario C., Ricci D., Camero M., Lorusso E., Elia G., Lavazza A., Martella V., Buonavoglia C. (2005). Virological and molecular characterization of a mammalian orthoreovirus type 3 strain isolated from a dog in Italy. Vet. Microbiol..

[205] Steyer A., Gutierrez-Aguire I., Kolenc M., Koren S., Kutnjak D., Pokorn M., Poljsak-Prijatelj M., Racki N., Ravnikar M., Sagadin M. (2013). High similarity of novel orthoreovirus detected in a child hospitalized with acute gastroenteritis to mammalian orthoreoviruses found in bats in Europe. J. Clin. Microbiol..

[206] Lewandowska D.W., Capaul R., Prader S., Zagordi O., Geissberger F.D., Kugler M., Knorr M., Berger C., Gungor T., Reichenbach J. (2018). Persistent mammalian orthoreovirus, coxsackievirus and adenovirus co-infection in a child with a primary immunodeficiency detected by metagenomic sequencing: A case report. BMC Infect. Dis..

[207] Lelli D., Moreno A., Steyer A., Naglic T., Chiapponi C., Prosperi A., Faccin F., Sozzi E., Lavazza A. (2015). Detection and Characterization of a Novel Reassortant Mammalian Orthoreovirus in Bats in Europe. Viruses.

[208] Johnson N., Vos A., Freuling C., Tordo N., Fooks A.R., Muller T. (2010). Human rabies due to lyssavirus infection of bat origin. Vet. Microbiol..

[209] Racey P.A., Hutson A.M., Lina P.H. (2013). Bat rabies, public health and European bat conservation. Zoonoses Public Health.

[210] Lumio J., Hillbom M., Roine R., Ketonen L., Haltia M., Valle M., Neuvonen E., Lahdevirta J. (1986). Human rabies of bat origin in Europe. Lancet.

[211] Fooks A.R., McElhinney L.M., Pounder D.J., Finnegan C.J., Mansfield K., Johnson N., Brookes S.M., Parsons G., White K., McIntyre P.G. (2003). Case report: Isolation of a European bat lyssavirus type 2a from a fatal human case of rabies encephalitis. J. Med. Virol..

[212] Dietz C., Von Helversen O., Nill D. (2007). Handbuch der Fledermäuse Europas und Nordwestafrikas.

[213] Serra-Cobo J., Amengual B., Abellan C., Bourhy H. (2002). European bat lyssavirus infection in Spanish bat populations. Emerg. Infect. Dis..

[214] Muller T., Cox J., Peter W., Schafer R., Johnson N., McElhinney L.M., Geue J.L., Tjornehoj K., Fooks A.R. (2004). Spill-over of European bat lyssavirus type 1 into a stone marten (*Martes foina*) in Germany. J. Vet. Med. B Infect. Dis. Vet. Public Health.

[215] Tjornehoj K., Fooks A.R., Agerholm J.S., Ronsholt L. (2006). Natural and experimental infection of sheep with European bat lyssavirus type-1 of Danish bat origin. J. Comp. Pathol..

[216] Phan T.G., Vo N.P., Bonkoungou I.J., Kapoor A., Barro N., O’Ryan M., Kapusinszky B., Wang C., Delwart E. (2012). Acute diarrhea in West African children: Diverse enteric viruses and a novel parvovirus genus. J. Virol..

[217] Yahiro T., Wangchuk S., Tshering K., Bandhari P., Zangmo S., Dorji T., Tshering K., Matsumoto T., Nishizono A., Soderlund-Venermo M. (2014). Novel human bufavirus genotype 3 in children with severe diarrhea, Bhutan. Emerg. Infect. Dis..

[218] Neuweiler G. (2000). The Biology of Bats.

[219] Hutterer R., Ivanova T., Meyer-Cords C., Rodrigues L. (2005). Bat Migrations in Europe: A Review of Banding Data and Literature.

[220] Rebelo H., Tarroso P., Jones G. (2010). Predicted impact of climate change on European bats in relation to their biogeographic patterns. Glob. Chang. Biol..

[221] Amorim F., Carvalho S.B., Honrado J., Rebelo H. (2014). Designing Optimized Multi-Species Monitoring Networks to Detect Range Shifts Driven by Climate Change: A Case Study with Bats in the North of Portugal. PLoS ONE.

[222] McCain C.M. (2007). Could temperature and water availability drive elevational species richness patterns? A global case study for bats. Glob. Ecol. Biogeogr..

[223] Sherwin H.A., Montgomery W.I., Lundy M.G. (2013). The impact and implications of climate change for bats. Mamm. Rev..

[224] Tuttle M.D., Adams R., Pedersen S. (2013). Threats to bats and educational challenges. Bat Evolution, Ecology, and Conservation.

[225] Boyles J.G., Cryan P.M., McCracken G.F., Kunz T.H. (2011). Conservation. Economic importance of bats in agriculture. Science.

[226] Olival K.J., Hosseini P.R., Zambrana-Torrelio C., Ross N., Bogich T.L., Daszak P. (2017). Host and viral traits predict zoonotic spillover from mammals. Nature.

[227] Calisher C.H., Childs J.E., Field H.E., Holmes K.V., Schountz T. (2006). Bats: Important reservoir hosts of emerging viruses. Clin. Microbiol. Rev..

[228] Kuemmerle T., Levers C., Erb K., Estel S., Jepsen M.R., Müller D., Plutzar C., Stürck J., Verkerk P.J., Verburg P.H. (2016). Hotspots of land use change in Europe. Environ. Res. Lett..

[229] Ramezani A., Chung S.J., Hutchinson S. (2017). A biomimetic robotic platform to study flight specializations of bats. Sci. Robot..

[230] Ifukube T., Sasaki T., Peng C. (1991). A blind mobility aid modeled after echolocation of bats. IEEE Trans. Biomed. Eng..

[231] Muise K.A., Menzies A.K., Willis C.K.R. (2018). Stress-induced changes in body temperature of silver-haired bats (*Lasionycteris noctivagans*). Physiol. Behav..

[232] Choisy M., Rohani P. (2006). Harvesting can increase severity of wildlife disease epidemics. Proc. Biol. Sci..

[233] Plowright R.K., Eby P., Hudson P.J., Smith I.L., Westcott D., Bryden W.L., Middleton D., Reid P.A., McFarlane R.A., Martin G. (2015). Ecological dynamics of emerging bat virus spillover. Proc. Biol. Sci..

[234] Paez D.J., Giles J., McCallum H., Field H., Jordan D., Peel A.J., Plowright R.K. (2017). Conditions affecting the timing and magnitude of Hendra virus shedding across pteropodid bat populations in Australia. Epidemiol. Infect..

[235] Sohayati A.R., Hassan L., Sharifah S.H., Lazarus K., Zaini C.M., Epstein J.H., Shamsyul Naim N., Field H.E., Arshad S.S., Abdul Aziz J. (2011). Evidence for Nipah virus recrudescence and serological patterns of captive *Pteropus vampyrus*. Epidemiol. Infect..

[236] Normile D. (2004). Infectious diseases. Mounting lab accidents raise SARS fears. Science.

[237] Normile D., Vogel G. (2003). Infectious diseases. Early indications point to lab infection in new SARS case. Science.

[238] Normile D. (2004). Infectious diseases. Second lab accident fuels fears about SARS. Science.

[239] Senior K. (2003). Recent Singapore SARS case a laboratory accident. Lancet Infect. Dis..

[240] Wurtz N., Papa A., Hukic M., Di Caro A., Leparc-Goffart I., Leroy E., Landini M.P., Sekeyova Z., Dumler J.S., Badescu D. (2016). Survey of laboratory-acquired infections around the world in biosafety level 3 and 4 laboratories. Eur. J. Clin. Microbiol. Infect. Dis..

[241] WHO (2021). WHO-Convened Global Study of Origins of SARS-CoV-2: China Part. https://www.who.int/publications/i/item/who-convened-global-study-of-origins-of-sars-cov-2-china-part.

[242] Boklund A., Hammer A.S., Quaade M.L., Rasmussen T.B., Lohse L., Strandbygaard B., Jorgensen C.S., Olesen A.S., Hjerpe F.B., Petersen H.H. (2021). SARS-CoV-2 in Danish Mink Farms: Course of the Epidemic and a Descriptive Analysis of the Outbreaks in 2020. Animals.

[243] Halfmann P.J., Hatta M., Chiba S., Maemura T., Fan S., Takeda M., Kinoshita N., Hattori S.I., Sakai-Tagawa Y., Iwatsuki-Horimoto K. (2020). Transmission of SARS-CoV-2 in Domestic Cats. N. Engl. J. Med..

[244] Sit T.H.C., Brackman C.J., Ip S.M., Tam K.W.S., Law P.Y.T., To E.M.W., Yu V.Y.T., Sims L.D., Tsang D.N.C., Chu D.K.W. (2020). Infection of dogs with SARS-CoV-2. Nature.

[245] Oreshkova N., Molenaar R.J., Vreman S., Harders F., Oude Munnink B.B., Hakze-van der Honing R.W., Gerhards N., Tolsma P., Bouwstra R., Sikkema R.S. (2020). SARS-CoV-2 infection in farmed minks, the Netherlands, April and May 2020. Eurosurveilliance.

[246] Oude Munnink B.B., Sikkema R.S., Nieuwenhuijse D.F., Molenaar R.J., Munger E., Molenkamp R., van der Spek A., Tolsma P., Rietveld A., Brouwer M. (2021). Transmission of SARS-CoV-2 on mink farms between humans and mink and back to humans. Science.

[247] Frutos R., Devaux C.A. (2020). Mass culling of minks to protect the COVID-19 vaccines: Is it rational?. New Microbes New Infect..

[248] Wang H., Wang F., Wang H., Zhao Q. (2020). Potential infectious risk from the pets carrying SARS-CoV-2. Travel Med. Infect. Dis..

[249] Schlottau K., Rissmann M., Graaf A., Schon J., Sehl J., Wylezich C., Hoper D., Mettenleiter T.C., Balkema-Buschmann A., Harder T. (2020). SARS-CoV-2 in fruit bats, ferrets, pigs, and chickens: An experimental transmission study. Lancet Microbe.

[250] Van Doremalen N., Schafer A., Menachery V.D., Letko M., Bushmaker T., Fischer R.J., Figueroa D.M., Hanley P.W., Saturday G., Baric R.S. (2018). SARS-Like Coronavirus WIV1-CoV Does Not Replicate in Egyptian Fruit Bats (*Rousettus aegyptiacus*). Viruses.

[251] Hall J.S., Knowles S., Nashold S.W., Ip H.S., Leon A.E., Rocke T., Keller S., Carossino M., Balasuriya U., Hofmeister E. (2020). Experimental challenge of a North American bat species, big brown bat (*Eptesicus fuscus*), with SARS-CoV-2. Transbound. Emerg. Dis..

[252] Wolfel R., Corman V.M., Guggemos W., Seilmaier M., Zange S., Muller M.A., Niemeyer D., Jones T.C., Vollmar P., Rothe C. (2020). Virological assessment of hospitalized patients with COVID-2019. Nature.

[253] Simmonds P., Aiewsakun P., Katzourakis A. (2019). Prisoners of war—Host adaptation and its constraints on virus evolution. Nat. Rev. Microbiol..

[254] Urbanowicz R.A., McClure C.P., Sakuntabhai A., Sall A.A., Kobinger G., Muller M.A., Holmes E.C., Rey F.A., Simon-Loriere E., Ball J.K. (2016). Human Adaptation of Ebola Virus during the West African Outbreak. Cell.

[255] Taubenberger J.K., Kash J.C. (2010). Influenza virus evolution, host adaptation, and pandemic formation. Cell Host Microbe.

[256] Li W., Zhang C., Sui J., Kuhn J.H., Moore M.J., Luo S., Wong S.K., Huang I.C., Xu K., Vasilieva N. (2005). Receptor and viral determinants of SARS-coronavirus adaptation to human ACE2. EMBO J..

[257] Plowright R.K., Parrish C.R., McCallum H., Hudson P.J., Ko A.I., Graham A.L., Lloyd-Smith J.O. (2017). Pathways to zoonotic spillover. Nat. Rev. Microbiol..

[258] Plowright R.K., Peel A.J., Streicker D.G., Gilbert A.T., McCallum H., Wood J., Baker M.L., Restif O. (2016). Transmission or Within-Host Dynamics Driving Pulses of Zoonotic Viruses in Reservoir-Host Populations. PLoS Negl. Trop. Dis..

[259] Voigt C.C., Phelps K.L., Aguirre L.F., Schoeman M.C., Vanitharani J., Zubaid A., Voigt C.C., Kingston T. (2016). Bats and Buildings: The Conservation of Synanthropic Bats. Bats in the Anthropocene: Conservation of Bats in a Changing World.

[260] Grange Z.L., Goldstein T., Johnson C.K., Anthony S., Gilardi K., Daszak P., Olival K.J., O’Rourke T., Murray S., Olson S.H. (2021). Ranking the risk of animal-to-human spillover for newly discovered viruses. Proc. Natl. Acad. Sci. USA.

[261] Beyer R.M., Manica A., Mora C. (2021). Shifts in global bat diversity suggest a possible role of climate change in the emergence of SARS-CoV-1 and SARS-CoV-2. Sci. Total Environ..

